# Supporting the Old but Neglecting the Young? The Two Faces of Ageism

**DOI:** 10.1037/dev0000903

**Published:** 2020-02-27

**Authors:** Christopher Bratt, Dominic Abrams, Hannah J. Swift

**Affiliations:** 1 Centre for the Study of Group Processes, School of Psychology, University of Kent

**Keywords:** ageism, discrimination, youth, social norms, European Social Survey

## Abstract

Ageism is the most prevalent form of prejudice and is experienced by both older and younger people. Little is known about whether these experiences are interdependent or have common origins. We analyze data from 8,117 older (aged 70 and over) and 11,647 younger respondents (15–29 years) in representative samples from 29 countries in the European Social Survey. Using multilevel structural equation modeling, we test the hypothesis that older people are less likely, and younger people more likely, to suffer age discrimination if they live in a country with stronger structural support for older people. We also test the hypothesis that although stronger social norm against age discrimination reduce age discrimination suffered by older people it does not inhibit discrimination against younger people. These hypotheses are supported, and the results underline the neglected problem of ageism toward youth. Findings highlight that strategies for reducing age prejudice must address ageism as a multigenerational challenge, requiring attention to intergenerational cohesion and resource distribution between ages.

Development across the life span involves not only biological and cognitive ageing, but also a changing social environment that responds differently as a function of how others perceive one’s age. In this article we consider how two aspects of the social environment—structural/economic resources, and social norms of discrimination, may affect the experiences of ageism among younger and older people. We propose that the same social environments are experienced differently by younger and older people, and that where conditions improve for older people, the conditions for younger people might well decline. Thus, our analysis is consistent with Bronfenbrenner’s (1979) ecological approach (see also Greene, Way, & Pahl, 2006), which emphasizes the role of subjective experience for understanding development within the wider social context and multiple levels of analysis.

The media, popular culture, and academic literature have all drawn attention to the problem of ageism. The academic literature tends to follow Butler’s (1975, p. 35) definition of ageism as “a process of systematic stereotyping of and discrimination against people because they are old” (Butler, 1969, 2005; Iversen, Larsen, & Solem, 2009; Nelson, 2005; North & Fiske, 2012; S. R. Levy & Macdonald, 2016). Yet, national surveys (Abrams & Houston, 2006; Bratt, Abrams, Swift, Vauclair, & Marques, 2018) and smaller-scale laboratory work (Garstka, Schmitt, Branscombe, & Hummert, 2004) show that younger people also experience ageism (Abrams, 2010; Hagestad & Uhlenberg, 2005; Snape & Redman, 2003). For instance, Bratt et al. (2018) found that across a large set of countries, younger people were more likely to report experiences of age discrimination than were older people. Surprisingly, psychology has largely neglected youth as being a major target of discrimination and, thus, has not paid much attention to the life span developmental implications of ageism, or the interdependence between younger and older people’s experiences.

An unanswered question is whether levels of age discrimination experienced by older and younger people are affected by common factors and whether measures to improve the conditions of older people might affect younger people negatively. We hypothesize that structural and ideological differences may account for variations in countries’ levels of age discrimination, and for differences in the extent to which younger and older people experience age discrimination. These hypotheses are tested using multilevel structural equation modeling (multilevel SEM) of data from representative samples from 29 countries in Round 4 of the European Social Survey (ESS).

## Modernization and Age Discrimination

A well-recognized account of why people may express ageism toward older people is provided by modernization theory. This sociological approach holds that modernization from a traditional and rural society into an urban and industrialized society will lead to a decline in the status of older people. Specifically, Cowgill (1974) proposed that industrialization and automation in modernized societies go along with falling birth rates and increased longevity, resulting in a higher proportion of older people and simultaneously a reduced demand for older workers’ skills. Cowgill suggested that the increased proportion of retired or unemployed older people and their dependency on younger working people result in a loss of status for older people.

Similar views on modernization have been proposed by social anthropologists (Schoenberg & Lewis, 2005) and psychologists (Branco & Williamson, 1982; Butler, 2009; Cuddy & Fiske, 2002; Nelson, 2005; North & Fiske, 2012). For instance, Nelson (2005) reasoned that modern demands for labor may emphasize youthful agility over mature experience and wisdom. Indeed, in research that asked North American participants to select which of two (equally qualified) candidates who could most maximize their organization’s performance (Abrams, Swift, & Drury, 2016), clear preference was shown for applicants whose profiles included positive stereotypes of younger age. These included being creative, good at learning new skills, and a quick decision maker. Deemed less hirable were applicants with profiles including positive older-age stereotypes, such as being good at settling arguments, understanding others’ viewpoints and being polite.

Theoretically, modernization results in a devaluation of older adults and might increase discrimination against this age group. However, research on age discrimination provides limited support to this notion. Studies comparing continents (North & Fiske, 2015) or countries across Europe (Vauclair et al., 2015) suggest relative positivity toward older adults in modernized societies. North and Fiske (2015) note that rises in population aging predict negative attitudes to older people, but that cultural individualism has the opposite effect—predicting positive attitudes to older people. Referring to research by Inglehart and colleagues (Inglehart & Baker, 2000; Inglehart, Norris, & Ronald, 2003), North and Fiske argue that once rapid industrialization has plateaued out in modernized societies, they develop increased tolerance and respect for the individual. Such tolerance and respect for the individual, North and Fiske reasoned, generates respect toward older people even within aging societies.

Increased tolerance in modernized societies is one possible explanation why older people experience less age discrimination than expected. However, modernization also involves structural changes. Specifically, modernization with increased prosperity has enabled many countries to provide financial and social support to older people. Such support might affect attitudes and age discrimination. These two alternative explanations—increased tolerance or structural support to older people—might at first sight appear complementary. However, they suggest different effects on age discrimination experienced among younger people.

If modernization improves the status of older people primarily by changing values and increasing tolerance in general, then such tolerance should probably also benefit younger people too. For example, an emphasis on tolerance and mutual respect could also heighten the appreciation of younger people’s energy and ingenuity, and their willingness to challenge old ideas. However, if modernization affects older people’s position primarily through structural factors, namely social and fiscal support to the older generation, it is not necessarily true that younger people will experience comparable benefits.

## Effects of Policies to Support Aging Populations

Cowgill (1974) identified four pillars of modernization: the development of economic technology, health technology, education, and urbanization. Three of these are captured by the Human Development Index (HDI), published by the United Nations (United Nations, 1990). The HDI is a composite based on economic strength as expressed by per capita income, the population’s education level, and life expectancy. Older people appear to hold higher social status in more modernized societies than in less modernized societies (North & Fiske, 2015; Vauclair et al., 2015). Consequently, and contrary to Cowgill’s assumptions, we would expect that high HDI scores should be associated with fewer reports of age discrimination among older people. For example, Bratt et al. (2018) observed low levels of age discrimination experienced among older respondents in Scandinavian countries and Switzerland, which have high levels of modernization. However, Bratt et al. also found that younger respondents in these countries reported experiencing higher levels of age discrimination. Therefore, we expect that high scores on the HDI should be associated with low levels of age discrimination experienced among older respondents, but high levels among younger respondents.

Given our contention that it is specifically increased structural support for older people that might be a basis for the positive (rather than negative) impact of modernization on older people, we use an indicator that will more directly reflect structural support for older people: the AgeWatch Index. This index has been developed by HelpAge International (www.helpage.org/global-agewatch) and Asghar Zaidi (2013), who writes:

The overarching purpose of the Global AgeWatch Index is to promote the development of policies and programs that will improve the quality of life and wellbeing of current and future generations of older people. [. . .] One of the strong motivations for the Index is the lack of age-disaggregated information across countries, leading to a poor understanding of the circumstances of older people in many countries (p. 5).

The AgeWatch Index uses data from the United Nations Department of Economic and Social Affairs, the World Bank, the World Health Organization, the International Labor Organization, UNESCO, and the Gallup World Poll to provide an overall score of how countries have used fiscal and social policies to improve the living conditions of older people (see Zaidi, 2013). Thus, the AgeWatch Index scores reflect structural differences between countries’ treatment and value of older people and is an excellent tool for research on ageism.

The HDI and AgeWatch Index have some overlap (see the online supplemental materials) and would be expected to be strongly related. However, the Age Watch Index includes additional elements such as the enabling environment for older people (social connections, physical safety, and access to public transport) that makes the specificity of the AgeWatch Index better attuned for testing our hypotheses regarding effects of structural support for older people.

We also expect the AgeWatch Index to predict age discrimination experienced among younger respondents, but differently than for older respondents. Income security and high-quality health-and-care services for older people systematically benefit the older population, but they are likely also to materially affect the younger population. For instance, policies that are designed to protect the pensions of older people necessarily draw on the resources generated by younger people. One example is the policy in the United Kingdom called “triple lock,” adopted in 2010 by the U.K. government to inflation-proof pensions (HM Treasury, 2010, p 4). The “triple lock” implies that the impact of austerity measures in the United Kingdom is lower for retired people than for most of the working population. We predict that a higher score on the AgeWatch Index score should be negatively associated with levels of age discrimination experienced by older people (Hypothesis 1a), but expect it to be positively associated with levels experienced among younger people (Hypothesis 1b).

## Effects of Normative Climate: The Role of Social Norms Against Age Discrimination

Modernization with increased standards of living, education, and life expectancy can affect attitudes to older people and older people’s experiences of age discrimination. Improved education within a country should probably also strengthen social norms against discrimination in general (Inglehart & Baker, 2000), and age discrimination specifically. Indeed, it is plausible that social norms can weaken or strengthen effects of structural factors. Therefore, it is important to consider both the role of social norms against age discrimination and the impact of structural factors (see Kinzig et al., 2013).

One might expect that people of all ages would experience low levels of age discrimination if they live in a country with strong social norms against ageism because such norms should provide a “motive to suppress prejudice” (Crandall, Eshleman, & O’Brien, 2002, p 359). Although this reasoning is consistent with research in various realms of intergroup relations (Crandall et al., 2002; Hogg & Abrams, 1988; Sherif, 1973; Stangor, Sechrist, & Jost, 2001; Zitek & Hebl, 2007), it seems inconsistent with the observation that some countries with low levels of old-age discrimination have high levels of young-age discrimination (Bratt et al., 2018). This contrast may, instead, be consistent with an alternative notion that even when people espouse ideology in favor of equality or have high levels of motivation to control prejudice, they apply these principles only to particular groups (a selective application of equality). In a representative survey of the U.K. population, Abrams, Houston, Van de Vyver, and Vasiljevic (2015) demonstrated that people apply the value of equality selectively—giving greater priority to equality for some groups than others. Specifically, respondents prioritized improvement of equal opportunities for groups that were viewed as more dependent (including older people) than for groups that posed symbolic threat to mainstream majority culture (such as gay people, Black or Muslim people). Given the different levels of discrimination experienced by older and younger people we anticipate that norms against age discrimination may be selectively applied too. Specifically, when people claim to be motivated to be unprejudiced based on age, they may implicitly be thinking only of older people, who are perceived to pose little threat to the status or power of other groups. Consequently, a norm against age discrimination is likely to be selectively applied for older people and, thus, fail to inhibit discrimination against younger people. We investigate this possibility by testing two hypotheses regarding the effects of social norms against age discrimination: country-level social norms against ageism will predict less frequent experiences of age discrimination among older respondents (Hypothesis 2a), but not among younger respondents (Hypothesis 2b).

Finally, we consider the combined effects of both the structural and normative contexts, and possible interaction effects. We expect that greater structural support and stronger antidiscrimination norms should both make a distinct contribution to the explanation of less frequent age discrimination experienced by older people (Hypothesis 3a). However, because of the selective application of social norms, only the AgeWatch Index is expected to predict age discrimination experienced by younger people (Hypothesis 3b), with higher scores on the AgeWatch Index being associated with more frequent age discrimination against this group.

Structural and normative factors may also interact. The effect of a general social norm against age discrimination may depend on how attentive governing authorities are toward the needs of specific age groups. We test for this possibility by investigating whether countries’ scores on the AgeWatch Index (representing social and fiscal support) moderate associations between social norms and experiences of age discrimination. We conduct this test among both older and younger respondents.

## Method

### Data

We used freely available data from the ESS Round 4, Edition 3 (2008), which contains no identifying information. It was not necessary to seek approval from the Institutional Review Board at the University of Kent. The ESS data were collected with computer aided personal interviews in 29 countries from the European region and Israel. Within each country, the ESS used strict probability methods to ensure that a national sample was representative for the country’s population aged 15 and over. Based on consensual age boundaries established in development work for the ESS, and consistent with the thresholds used elsewhere in the ESS survey (that refer to “people over 70” and “people in their 20s”), we operationalized the sample of “older” respondents as being aged 70 or more (after dropping a respondent coded as 123 years old in the ESS data): *n* = 8,117, age span from 70 to 105, *M*_age_ = 76.9, *SD* = 5.41, 60% female. We defined the sample of “younger” respondents as being below 30 years: *n* = 11,647, age span from 15 to 29, *M*_age_ = 22.7, *SD* = 4.17, 51% female.

The ESS data had very few missing responses. Nearly all older (97%) and nearly all younger respondents (98%) had complete data, see the online supplemental materials for details. Since we used full information maximum likelihood estimations, which will include cases with partly missing data, only five respondents 70 years or older and only three respondents between 15 and 29 years were dropped from the analyses. The large representative samples from 29 countries (with overall sample sizes of more than 8,000 older respondents and more than 11,500 younger respondents) provided a solid basis for the current analyses.

### Measures

#### Experiences of age discrimination

Experiences of age discrimination were assessed with three items. In the English version, these items read: (a) “Please tell me how often, in the past year, anyone has shown prejudice against you or treated you unfairly because of your age?” (b) “How often, if at all, in the past year have you felt that someone showed you a lack of respect because of your age, for instance by ignoring or patronizing you?” (c) “How often in the past year has someone treated you badly because of your age, for example by insulting you, abusing you or refusing you services?” The three items on prejudice or unfair treatment, lack of respect, and being treated badly because of age were all assessed using a 5-point scale from 0 to 4, labeling only the extremes *never* (0) and *very often* (4), as well as a “Don’t know” option that was coded as missing data. The three items on experiences of age discrimination provide a relatively brief measurement, with items selected from multiple items (Vauclair, Abrams, & Bratt, 2010). The limitation of having few items is compensated for by the sample sizes available in the ESS. Furthermore, earlier research has shown that these items in the ESS have a high degree of measurement invariance across countries and languages, and across age. Measurement invariance is satisfied even when tested across age as a continuous variable (Bratt et al., 2018).

**Figure 1 fig1:**
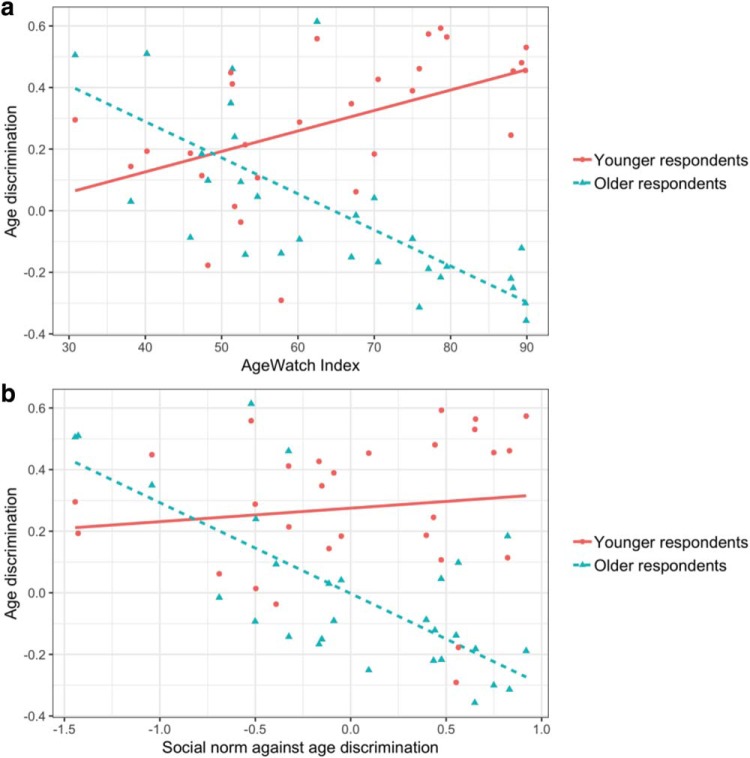
Scatterplots (with regression lines) of country-level means of experiences of age discrimination and (a) countries’ scores on the AgeWatch Index or (b) country-level social norms against age discrimination. The plots show separate results for younger and older respondents in each country. See the online supplemental materials for each country’s position on the two plots.

Statistical analyses in the present research used the original, 5-point ordinal items. Plots in Figure 1 and various plots in the online supplemental materials used composite scores, the mean of all three items. We note that these composite scores had substantial country-level variation: the intraclass correlation was .09 for older respondents and .05 for younger respondents, meaning that 9 and 5% of the overall variations in composite scores were at the country level.[Fig-anchor fig1]

#### The AgeWatch Index

We used countries’ scores on the AgeWatch Index (www.helpage.org/global-agewatch) as a country-level predictor, reflecting fiscal and social support for a country’s older population. We used the AgeWatch Index data for 2013, the earliest data available. Asghar Zaidi, the scientific consultant for HelpAge International in the development of the AgeWatch Index, describes the indicators in detail and groups them into four domains (Zaidi, 2013, pp. 9–14), with equal weight to each domain:

1 *Income security for older people*: Pension income coverage (40% weight for the domain score); poverty rate in old age (20%); relative welfare of older people (20%); GDP per capita (20%).
2 *Health status*: Life expectancy at 60 (40% weight for the domain score); healthy life expectancy at 60 (40%); psychological wellbeing (20%).
3 *Employment and education*: Employment of older people (50% weight for the domain score); education status of older people (50%).
4 *Enabling environments for older people*: Social connections (25% weight for the domain score); physical safety (25%); civic freedom (25%); access to public transport (25%).

To reduce computational problems in multilevel analyses because of high variance, we first centered scores on the AgeWatch Index and then divided them by 5, which resulted in a scale from −6.59 to 5.23. The original country scores are shown in the online supplemental materials.

#### Social norms

Sociological theory (e.g., Mead, 1934) suggests that social norms exert an effect on individual behavior because of their internalization as personal norms. Being internalized, personal norms are not dependent on social sanctions to exert an effect on behavior (Schwartz, 1977), but they continue to reflect societal norms and values (Abrams & Hogg, 1990; Allport, 1954; Bratt, 1999; Sherif, 1973). Because of the link between personal and social norms, it is possible to develop measurements of country differences in overall norms against age discrimination by aggregating individually expressed social norms (e.g., Manning, Newman, Valliere, Wang, & Lawson, 2001). The ESS survey included two items assessing personal norms against age prejudice, one for internal and one for external motivations: (a) “Please tell me how important it is for you to be unprejudiced against people of other age groups.” (b) “Please tell me how important it is for you to be seen as being unprejudiced against people of other age groups.” Both items used an 11-point response scale, with the extreme answers labeled *not at all important* and *extremely important*, as well as a *don’t know* option, which was treated as missing data. These items were drawn from previous measures developed by Plant and Devine (1998), and also by Abrams et al. (2015). The two items were strongly correlated (overall *r* = .62, *p* < .001) and we computed a mean score of the two items for each respondent and then grand-mean centered this variable. We then computed country-level means of the composite scores. We deliberately excluded members of the target group when estimating country-level means. Thus, we estimated two variables for country-level social norms. We used the social norm expressed among respondents aged between 15 and 49 as a predictor of age discrimination experienced by respondents aged 70 years or older. Conversely, we used the social norm expressed among respondents aged between 40 and 105 years to predict age discrimination experienced by respondents aged under 30. The two assessments of country-level norms were nearly identical (*r* = .97).

#### Individual-level predictors

Individual-level predictors of experiences of age discrimination were age and gender. We centered age within each age group, and we coded gender as male = 0 and female = 1.

#### Descriptive statistics

The online supplemental materials show descriptive statistics, the raw data are available from the ESS server (www.europeansocialsurvey.org).

### Analytical Strategy

We used R 3.4.3 (R Core Team, 2016) and Stata 15 for data management, and the R package knitr (Xie, 2016) to compile the online supplemental materials. We used the ggplot2 package (Wickham, 2009) in R for descriptive graphics. Multilevel SEM was conducted with M*plus* 8.3 (Muthén & Muthén, 1998–2017) and integrated with R using MplusAutomation (Hallquist & Wiley, 2018). We used the R package kableExtra (Zhu, 2019) to develop tables, using code to automatically collect results from M*plus*’ output files.

Multilevel SEM in M*plus* used either its default estimator (maximum likelihood with robust standard errors, MLR) or Bayesian estimations. Bayesian multilevel estimations are more conservative than MLR estimations (Stegmueller, 2013), providing a test of the robustness of the findings obtained with maximum likelihood estimations in particular when multilevel analyses include few clusters (countries in this case). Maximum likelihood and Bayesian estimations may not give identical results and some scholars recommend considering both approaches and to compare their results to increase the robustness of research (Lele & Dennis, 2009).

Bayesian estimations used two Markov Chain Monte Carlo (MCMC) chains and 1,500,000 iterations for each chain (without thinning, see Link & Eaton, 2012). Bayesian estimations used noninformative priors, meaning that the estimations were data-based and not influenced by any theory-based assumptions of how strong the associations would be.

The multilevel models estimated experiences of age discrimination as a latent variable both at the individual and at the country level. We estimated models for younger and older respondents separately and did not force factor loadings or thresholds to be equal across the two age groups. At the individual level, factor loadings for the indicators were similar, consistent with the high level of measurement invariance demonstrated by Bratt et al. (2018).

Four different multilevel SEM models were estimated for both older and younger respondents separately, testing the AgeWatch Index as the only predictor (Model 1), social norms as the only predictor (Model 2), and an additive model with both the AgeWatch Index and social norms as predictors (Model 3). We also tested for possible interaction effects between the AgeWatch Index and social norms (Model 4). Age within an age group and gender (female) were predictors at the individual level, with limited explanatory power in both age groups, though age was moderately associated with less frequent experiences of age discrimination among younger respondents.

We evaluated model fit with fit indices available in the specific analyses applied. For estimations with maximum likelihood, we used the Akaike Information Criteria (AIC) and the Bayesian Information Criteria (BIC). These fit indices allow for a comparison of nonnested models and reflect the models’ ability to explain the dependent variable relative to the model’s parsimony (punishing for model complexity, the BIC more than the AIC). A lower value is preferred.

Analyses with Bayes estimations gave a Posterior Predictive P-Value (PPP) as a fit estimate. A perfectly fitting model will have a PPP at .50. The estimation of PPP in M*plus* is based on the chi-square test and any PPP above .05 is considered “nonsignificant,” that is, indicating that the model fits the data. The current analyses had large sample sizes, resulting in a powerful test for a fit index based on the chi-square, easily estimating trivial deviations between model-implied moments and moments in the data to be “statistically significant”—in particular among younger respondents because of their sample size of more than 11,500. In addition to the PPP, the online supplemental materials report the potential scale reduction for the four chains used in Bayesian estimations (should indicate no difference by being close to 1, i.e., below 1.05 for iterations after the initial burn-in). We also inspected Bayesian plots for signs of convergence problems. Finally, we reran all Bayesian analyses doubling the number of iterations (using four chains with 3,000,000 Bayesian iterations) and with a different Bayesian seed number, testing for any differences (bias) across the two Bayesian estimations.

## Results

### Structure and Social Norms as Predictors of Age Discrimination

As anticipated, the HDI and AgeWatch Index were highly related (*r* > .89 regardless of whether measured in 2008 or 2013). As it was not statistically defensible to use both indices in multilevel models, we focus here on the analyses using the AgeWatch Index. However, comparable results are attained when using the HDI. The online supplemental materials show results for the HDI and also associations between the GINI index (equality within countries) and experiences of age discrimination. In more equal societies, relative to less equal societies, older respondents experienced age discrimination less often whereas younger respondents experienced age discrimination more often.

Table 1 shows results of multilevel SEM models for both older and younger respondents. Differences were notable at the country level. The AgeWatch Index (Model 1) was strongly associated with country-level differences in experiences of age discrimination, both among older (*R*^2^ = .60) and among younger respondents (*R*^2^ = .50). Supporting the hypotheses on structural effects of financial and social support to older people, the AgeWatch Index predicted *less frequent* experiences of age discrimination among older respondents (*b* = −0.22 [95% confidence interval, CI [−0.29, −0.15]], β = −.76; consistent with Hypothesis 1a), but *more frequent* among younger respondents (*b* = 0.21 [0.13, 0.29], β = .71; consistent with Hypothesis 1b). These differences between younger and older respondents are illustrated in Figure 1a. Further details on associations between the AgeWatch Index and experiences of age discrimination in the various countries are provided in the online supplemental materials (Figure S3a and Table S7).[Table-anchor tbl1]

Social norms against age discrimination (Model 2) were a substantial and negative predictor of age discrimination experienced by older respondents (*b* = −1.04 [−1.45, −0.63], β = −.72; consistent with Hypothesis 2a). However, norms against age discrimination had a weak and *positive* association with experiences of age discrimination among younger respondents (see also Figure 1b). The confidence interval for younger respondents’ experiences of age discrimination included both positive and negative values (*b* = 0.24 [−0.29, 0.78], β = .16) and, thus, both a weak positive and weak negative association were compatible with the data. Evidently, the analysis failed to indicate a significant association between social norms and age discrimination experienced among younger respondents, consistent with Hypothesis 2b. Further details are available in the online supplemental materials (Figure S3b and Table S8).

One assumption motivating the present research was the belief that structural factors favoring older people can explain why modernization has not led to the expected overt devaluation of older people and that such structural factors may prove more important than ideological factors. Supporting this assumption, the model with only AgeWatch Index as a country-level predictor appeared moderately superior to the model with only social norms as the country level predictor. Using the AgeWatch Index as the single country-level predictor explained more variance in older people’s experiences of age discrimination (*R*^2^ = .60) than did social norms (*R*^2^ = .51), and both the AIC and the BIC measures of model fit favored the AgeWatch Index (Model 1) over social norms (Model 2). Findings for younger respondents’ experiences of discrimination revealed that for this age group, only structural factors and not social norms had a substantial association with country-level age discrimination (AgeWatch Index, *R*^2^ = .50; social norms, *R*^2^ = .02).

### Additive and Interaction Effects

When the analysis included both the AgeWatch Index and social norms against age discrimination as country-level predictors (Model 3), both these variables remained negative predictors of age discrimination experienced among older respondents (*b* = −0.16 [−0.23, −0.09], β = −.55; *b* = −0.59 [−1.01, −0.16], β = −.41), consistent with the Additive Hypothesis 3a. Only the AgeWatch Index in Model 3 was a strong predictor of younger respondents’ experiences of age discrimination, predicting more frequent experiences of age discrimination (*b* = 0.27 [0.17, 0.36], β = .47), consistent with the Additive Hypothesis 3b. This additive model also estimated social norms to be a moderately negative predictor, however the credibility interval included positive values (*b* = −0.47 [−1.01, 0.07]).

The final model (Model 4) included the country-level interaction effect between the AgeWatch Index and social norms (see Table S10 in the online supplemental materials for detailed results). No interaction effect was indicated for older respondents (*b* = −0.01 [−0.09, 0.07]). However, among younger respondents, the analysis with maximum likelihood estimations indicated an interaction effect (*b* = 0.14 [0.05, 0.23]), suggesting that structural support to older people may inhibit or prevent antiageist social norms from reducing age discrimination toward younger people.

### Tests With Bayesian Estimations

To test the robustness of the earlier findings, we used a more conservative method, rerunning all models with Bayesian estimations. The Bayesian estimations supported the earlier findings in the first three models. As expected, point estimates for country-level regression weights tended to be lower in Bayesian estimations, but the Bayesian estimations also suggested less uncertainty (the 95% credibility intervals for Bayesian estimations were narrower than the 95% confidence intervals obtained with MLR estimations, see the online supplemental materials for details). In the interaction model (Model 4), the Bayesian estimation of responses from younger respondents resulted in a credibility interval that included both positive and negative values (*b* = 0.06 [−0.02, 0.14]), indicating greater uncertainty about a possible interaction effect.

## Discussion

Population aging and increasing longevity have stimulated awareness of prejudice and discrimination toward older adults, and intentions to reduce such prejudice (Officer et al., 2016; WHO, 2011). Curiously, despite being very prevalent, younger people’s experiences of ageism have largely been overlooked both in the research literature and by policymakers. For instance, earlier research has investigated how modernization is associated with the societal status of older people, but has ignored the equally interesting question how it bears on the status of younger people. The analysis presented here addresses this significant gap in research by exploring the extent to which older and younger people’s experiences of age discrimination might be interdependent. Our analysis also offers an account that representatively captures the experiences of older and younger people across almost an entire continent.

We assumed that the structural effects of modernization on age discrimination would be through policies that benefit and protect older people (reflected by the AgeWatch Index). Our assumption that modernization would predict structural support for older people was corroborated by the very high shared variance (80%) between the HDI and the AgeWatch Index. We were then able to test the specific hypothesis that structural support to older people should have different effects on the age discrimination experienced among older and younger people. We also note that findings were similar if we used the HDI instead of the AgeWatch Index as country-level predictor.

Multilevel SEM confirmed that the AgeWatch Index predicted *less frequent* experiences of age discrimination among older respondents, but *more frequent* among younger respondents. This is a completely novel, and highly consequential finding because we have established that national policies to support older people can result in substantially greater discrimination against younger people, an age group that is already more vulnerable to other forms of discrimination (e.g., discrimination based on ethnicity, see Abrams & Houston, 2006).

Multilevel SEM also shed new light on how psychological processes, specifically the normative climate within a society, may combine with structural conditions to the advantage or disadvantage of older and younger people. The analyses indicated that stronger social norms against age discrimination predicted lower discrimination only against older people. Social norms against age discrimination had a weak and positive association with younger respondents’ experiences of age discrimination in one model (Model 2), and a weak and negative association in another (Model 3). In both these models, the confidence intervals included both negative and positive values, corroborating the hypothesis that social norms against age discrimination have no substantial relationship with age discrimination experienced among younger respondents. The cross-national finding that norms against age discrimination explained levels of age discrimination experienced among older, but not among younger people extends the more general evidence that people do not apply equality values equally to all groups (Abrams et al., 2015).

The present data provided some support for the proposition that structural and normative contexts would have an interactive effect on experiences of discrimination among younger respondents. A traditional analytic method (maximum likelihood estimation) gave a confidence interval that excluded zero. The interaction indicated that structural support to older people moderated the association between antiageist norms and levels of age discrimination experienced by younger people. Specifically, in countries where older people were better supported, strong antiageism norms were associated with higher levels of discrimination experienced by younger people. However, we interpret this cautiously because the credible interval in the much more conservative Bayesian analysis marginally encompassed zero. We attribute the less certain result with Bayesian analysis to the limited number of countries in this research.

### An Integrated Perspective on Age

Our research points to the need for an integrated perspective that includes both older and younger members of society when studying ageism (Hagestad & Uhlenberg, 2005; Roberts, 2006), and underpins the value of a more ecologically sensitive approach (Bronfenbrenner, 1979). Restricting the challenge of reducing ageism only to the situation of older people might itself be implicitly ageist, particularly given the higher prevalence of age discrimination against younger people indicated by European data.

An integrated perspective on discrimination across age can also incorporate middle-aged people as a third group of interest. For the present, we decided not to include analyses of age discrimination experienced by middle-aged respondents. Middle-aged people have more varied life circumstances compared with the more distinctive younger and older categories, who are closer to birth and death, respectively. Moreover, preliminary comparisons of age discrimination experienced among older (70 years and more) and middle-aged respondents (40 to 55 years old) in the ESS confirm that age discrimination experienced among middle-aged are notably less frequent than among older people.

### Strengths and Limitations

This research has relied on cross-sectional data, but we see the findings as robust. The theoretical foundation of the research is strong, and we used advanced statistical methods. Furthermore, the data in the ESS are of high quality, with representative and large samples of younger and older respondents in each of the 29 countries.^1^ It is obviously important to consider the possibility of reversed causality. A reversed causality seems unlikely because scores on the AgeWatch Index could not feasibly have been affected by reports in the ESS on age discrimination. Similarly, because we assessed social norms in one age group and experiences of age discrimination in the other it seems unlikely that the causal direction would go from discrimination to norms (e.g., that older people’s norms would be caused by younger people’s experiences).

One limitation of the present research is that it does not apply to the experiences of children and teenagers below 15 years, because of the restriction on age in the ESS. However, given that the experience of these children is largely confined to the closest family and institutional settings where trained adults have the mandate to supervise children (kindergarten and school), age-based discrimination of children is primarily at an institutional level and perhaps not experienced as discrimination. Only when people leave compulsory education can age discrimination be directly compared across ages. Similarly, the current findings are less likely to be applicable to the experiences of the very oldest people, the “old-old” aged 85 or more, because they are likely to be undersampled. We used the weighting variable available in the ESS to counteract selection bias, but even with weighting included, the ESS data may have a nonrandom selection bias among the very oldest. Many of the very oldest will have health issues and be in various forms of institutional care such as nursing homes and are difficult to access with surveys. The inaccessibility of data from the very oldest is not specific to this research but characterizes most of the research on older people.

A limitation in the ESS measurements is that it included only three broad items to assess experiences of discrimination. These three items are representative of a wider and more extensive set (Vauclair et al., 2010), but it would be useful if future research could investigate whether, for example, the structural and normative protections associated with the AgeWatch Index and normative climate are restricted to particular aspects of discrimination such as hostile or benevolent ageism (Cary, Chasteen, & Remedios, 2017), and whether these differ for younger and older people. More generally, the present research highlights that the same structural and normative context can mean quite different things for the experiences of ageism among younger and older people. Thus, in terms of life span development and for intergenerational relations more generally, these contextual factors may have changing implications for the self-concept and the value that people attach to old age and the ageing process. In turn, this could have implications for stereotype embodiment and vulnerability to stereotype threat effects (B. Levy, 2009; Lamont, Swift, & Abrams, 2015).

### Implications for Policy

An important policy implication of the present research is that interventions that are designed to enhance the living conditions of older adults should also consider whether there may be undesired negative effects on other age groups. For instance, strategies that overtly benefit older people, such as creating age-friendly workplaces or communities, might inadvertently heighten younger people’s experiences of age discrimination because they feel deprioritized or relatively neglected. For example, younger people may sometimes be patronized in the workplace (Duncan & Loretto, 2004). Moreover, national or global campaigns to reduce ageism such as those proposed by WHO (Officer et al., 2016), which aim to raise awareness of ageism and enhance social norms against ageism, should consider ways of ensuring that these strategies also embrace younger people’s experiences of ageism, particularly in countries that already provide relatively good support for older adults.

More broadly, the present findings have implications for more complex intersectional intergroup relationships. Given that strategies to address disadvantages experienced by one target group may miss important disadvantages experienced by others, the possibility that discrimination can arise through neglect rather than deliberate or actively discriminatory policies is clearly pertinent to many contexts beyond age. Moreover, although both older and younger people may encounter multiple stigma, some evidence indicates that ageism against younger people may be compounded by other group memberships they hold (Abrams & Houston, 2006). For example, young people are overrepresented among immigrants and refugees (UNCHR, 2019). A structural and normative climate that strongly favors older people may mean that both resources and inhibitions on discrimination are attenuated in the case of these groups. While it would be absurd to propose that policies should withdraw support from older people, it is equally problematic not to heed the possibility that risks of discrimination against other groups may be particularly heightened if their members tend to be young.

### Conclusion

The research presented extends previous research on ageism toward old and young (e.g., Abrams, 2010; Bratt et al., 2018; North & Fiske, 2012) by showing empirically how younger and older adults’ experiences of ageism are interrelated as a function of the social context. It broadens the focus of ageism research by placing equal emphasis on young-ageism and old-ageism. We have established that the presence of structural support for older people is reflected in lower frequency of age discrimination experienced by this age group but seems to contribute to increased experiences of age discrimination among younger people. Further, we have found that social norms against age discrimination are associated with low levels of age discrimination experienced among older people, but not among younger people. This research also suggests that modernization affects older and younger people differently and that such effects are associated with the different levels of structural and normative support for the older population.

These findings offer new insights into the nature of and influences on ageism, and particularly highlight the need to pursue research into ageism against youth and not just old age. Tackling both forms of ageism and finding ways to improve intergenerational cohesion and support will become increasingly pressing tasks as life expectancy increases and societies become ever more age diverse.

## Supplementary Material

10.1037/dev0000903.supp

## Figures and Tables

**Table 1 tbl1:** Multilevel SEM of Experiences of Age Discrimination, Separate Results for Older and Younger Respondents

Parameters	Older respondents		Younger respondents	
	Estimate	95% CI	Estimate	95% CI
Model 1				
Individual level				
Loading prejudice	1.00	[1.00, 1.00]	1.00	[1.00, 1.00]
Loading respect	1.78	[1.44, 2.12]	2.01	[1.65, 2.38]
Loading treated	1.79	[1.46, 2.13]	1.45	[1.22, 1.68]
Female	0.27	[0.05, 0.49]	0.12	[0.01, 0.24]
Age	0.01	[−0.01, 0.03]	−0.07	[−0.09, −0.05]
*R*^2^	0.00		0.02	
Country level				
Loading prejudice	1.00	[1.00, 1.00]	1.00	[1.00, 1.00]
Loading respect	1.85	[1.32, 2.39]	1.40	[1.04, 1.77]
Loading treated	2.11	[1.63, 2.58]	0.44	[0.13, 0.75]
AgeWatch Index	−0.22	[−0.29, −0.15]	0.21	[0.13, 0.29]
*R*^2^	0.60		0.50	
Model fit				
AIC	35887.42		69498.93	
BIC	36034.29		69653.45	
Model 2				
Individual level				
Loading prejudice	1.00	[1.00, 1.00]	1.00	[1.00, 1.00]
Loading respect	1.78	[1.44, 2.12]	2.01	[1.65, 2.38]
Loading treated	1.79	[1.46, 2.13]	1.45	[1.22, 1.68]
Female	0.27	[0.05, 0.49]	0.12	[0.01, 0.24]
Age	0.00	[−0.01, 0.03]	−0.07	[−0.09, −0.05]
*R*^2^	0.00		0.02	
Country level				
Loading prejudice	1.00	[1.00, 1.00]	1.00	[1.00, 1.00]
Loading respect	1.84	[1.32, 2.37]	1.41	[1.04, 1.77]
Loading treated	2.08	[1.61, 2.55]	0.45	[0.14, 0.75]
Social norm	−1.04	[−1.45, −0.63]	0.24	[−0.29, 0.78]
*R*^2^	0.51		0.02	
Model fit				
AIC	35892.20		69517.26	
BIC	36039.07		69671.78	
Model 3				
Individual level				
Loading prejudice	1.00	[1.00, 1.00]	1.00	[1.00, 1.00]
Loading respect	1.78	[1.44, 2.12]	2.01	[1.65, 2.38]
Loading treated	1.79	[1.46, 2.13]	1.45	[1.22, 1.68]
Female	0.27	[0.05, 0.49]	0.12	[0.01, 0.24]
Age	0.01	[−0.01, 0.03]	−0.07	[−0.09, −0.05]
*R*^2^	0.00		0.02	
Country level				
Loading prejudice	1.00	[1.00, 1.00]	1.00	[1.00, 1.00]
Loading respect	1.84	[1.31, 2.38]	1.40	[1.04, 1.77]
Loading treated	2.10	[1.62, 2.57]	0.44	[0.13, 0.75]
AgeWatch Index	−0.16	[−0.23, −0.09]	0.27	[0.17, 0.36]
Social norm	−0.59	[−1.01, −0.16]	−0.47	[−1.01, 0.07]
*R*^2^	0.72		0.56	
Model fit				
AIC	35880.85		69496.99	
BIC	36034.71		69658.86	
*Note*. SEM = structural equation modeling; CI = confidence interval; AIC = Akaike information index; BIC = Bayesian information index. Prejudice refers to the item “treated with prejudice because of age.” Respect refers to the item “lack of respect because of age.” Treated refers to the item “treated badly because of age.”				

## Footnotes

1 See http://www.europeansocialsurvey.org/methodology/ess_methodology/sampling.html and http://www.europeansocialsurvey.org/docs/round8/methods/ESS8_sampling_guidelines.pdf.
