# Supplementary material for: Supporting the Old but Neglecting the Young? The Two Faces of Ageism
Source: Dev Psychol. 2020 Feb 27;56(5):1029–39. doi: 10.1037/dev0000903 (PMC7144460; doi:10.1037/dev0000903)
Supplement: Supplementary file 1 [file dev0000903_Supplemental-Materials.pdf]

Supporting the Old but Neglecting the Young? The Two Faces of Ageism – Supplemental Materials

Contents

Introduction 4

The Human Development Index and the AgeWatch Index 4

Descriptive Statistics 5

Plots using the Human Development Index and the GINI Index 8

Plots using AgeWatch Index Scores and Social Norms 10

Comparing Four Groups of Countries 13

Multilevel SEM: Maximum Likelihood Estimations 14

Multilevel SEM: Bayesian Estimations 20

Tests of Convergence of Bayesian Analyses 26
- Bayesian Plots . . . 26
- Doubling the Number of Bayesian Iterations . . . 27
- Plots of Bayesian Parameters . . . 31

Additional Documentation 56
- Examples of Input for Multilevel SEM . . . 56

References 58

Supplemental materials to Bratt, Abrams, and Swift (2020). Supporting the old but neglecting the young? The two faces of ageism, *Developmental Psychology*

List of Tables

S1 Frequences of the three age discrimination items, in percents 5
S2 Correlation table for country-level composite scores of age discrimination and predictors (N = 29, p-values in parentheses) 5
S3 Patterns of missing data (x = not missing) 6
S4 Countries' scores on the AgeWatch Index 7
S5 ISO codes and country names 8
S6 The Human Development Index as country-level predictor of experiences of age discrimination, maximum likelihood estimations (separate results for older and younger respondents) 15
S7 Model 1. The AgeWatch Index as country-level predictor of experiences of age discrimination, maximum likelihood estimations (separate results for older and younger respondents) 16
S8 Model 2. Social norms as country-level predictor of experiences of age discrimination, maximum likelihood estimations (separate results for older and younger respondents) 17
S9 Model 3. The AgeWatch Index and social norms as country-level predictors of experiences of age discrimination, maximum likelihood estimations (separate results for older and younger respondents) 18
S10 Model 4. The AgeWatch Index, social norms, and their interaction as country-level predictors of experiences of age discrimination, maximum likelihood estimations (separate results for older and younger respondents) 19
S11 Model 1 (Bayesian estimations). The AgeWatch Index as country-level predictor of experiences of age discrimination (separate results for older and younger respondents) 22
S12 Model 2 (Bayesian estimations). Social norms as country-level predictor of experiences of age discrimination (separate results for older and younger respondents) 23
S13 Model 3 (Bayesian estimations). The AgeWatch Index and social norms as country-level predictors of experiences of age discrimination (separate results for older and younger respondents) 24
S14 Model 4 (Bayesian estimations). The AgeWatch Index and social norms as well as their interaction as country-level predictors of experiences of age discrimination (separate results for older and younger respondents) 25
S15 Model 1, differences (bias) for point estimates with either 1.5 million or 3.0 million Bayesian iterations 27
S16 Model 2, differences (bias) for point estimates with either 1.5 million or 3.0 million Bayesian iterations 28
S17 Model 3, differences (bias) for point estimates with either 1.5 million or 3.0 million Bayesian iterations 29
S18 Model 4, differences (bias) for point estimates with either 1.5 million or 3.0 million Bayesian iterations 30

List of Figures

S1 Countries' scores on the AgeWatch Index . . . 6
S2 Comparisons of country-level scores for age discrimination experienced among younger and older respondents and (a) the HDI or (b) the GINI. . . . 9
S3 Country differences in age discrimination experienced by older and younger respondents, dependent on AgeWatch Index scores and social norms against age discrimination. . . . 11
S4 Country differences in age discrimination experienced by older and younger respondents, dependent on AgeWatch Index scores and social norms against age discrimination. Portugal, Cyprus, and Czechia dropped. . . . 12
S5 Comparison of four groups of countries, dependent on scores on the AgeWatch Index (high or low) and social norms (strong or weak) . . . 13
S6 The AgeWatch Index and social norms against age discrimination predicting experiences of age discrimination: (a) Older respondents, MLR estimations, (b) Older respondents, Bayesian estimations, (c) Younger resondents, MLR estimations, (d) Younger respondents, Bayesian estimations . . . 21
S7 Older respondents, Model 1, trace plots . . . 32
S8 Older respondents, Model 1, distribution plots . . . 33
S9 Older respondents, Model 1, autocorrelation plots . . . 34
S10 Older respondents, Model 2, trace plots . . . 35
S11 Older respondents, Model 2, distribution plots . . . 36
S12 Older respondents, Model 2, autocorrelation plots . . . 37
S13 Older respondents, Model 3, trace plots . . . 38
S14 Older respondents, Model 3, distribution plots . . . 39
S15 Older respondents, Model 3, autocorrelation plots . . . 40
S16 Older respondents, Model 4, trace plots . . . 41
S17 Older respondents, Model 4, distribution plots . . . 42
S18 Older respondents, Model 4, autocorrelation plots . . . 43
S19 Younger respondents, Model 1, trace plots . . . 44
S20 Younger respondents, Model 1, distribution plots . . . 45
S21 Younger respondents, Model 1, autocorrelation plots . . . 46
S22 Younger respondents, Model 2, trace plots . . . 47
S23 Younger respondents, Model 2, distribution plots . . . 48
S24 Younger respondents, Model 2, autocorrelation plots . . . 49
S25 Younger respondents, Model 3, trace plots . . . 50
S26 Younger respondents, Model 3, distribution plots . . . 51
S27 Younger respondents, Model 3, autocorrelation plots . . . 52
S28 Younger respondents, Model 4, trace plots . . . 53
S29 Younger respondents, Model 4, distribution plots . . . 54
S30 Younger respondents, Model 4, autocorrelation plots . . . 55

Introduction

These supplemental materials describe similarities and differences between the HDI and the AgeWatch Index, show descriptive statistics and report details of analyses summarized in the article. Analyses using the Human Development Index (HDI) as a predictor of age discrimination are also included. Toward the end we show Mplus input with maximum likelihood estimations and with Bayesian estimations.

This document is compiled with R and RStudio, using the `knitr` package (Xie, 2016). Mplus analyses were integrated with R using the `MplusAutomation` package (Hallquist and Wiley, 2018). Tables were developed with `kableExtra` (Xhu, 2019), plots with `ggplot2` (Wickham, 2009).

The Human Development Index and the AgeWatch Index

The Human Development Index (HDI) is a measure of countries' average achievement in key dimensions of human development, originally focusing on three dimensions: (1) standard of living, as measured by the GDP per capita, adjusted for purchasing power parity, (2) education level, and (3) life expectancy at birth. We used HDI data available in the Human Development Reports (http://hdr.undp.org/en/data#).

Analyses used HDI data for 2008 (the year the ESS data in Round 4 were collected). In 2008, the HDI did not yet incorporate social equality as a fourth dimension in the computation of the index. We add a plot with GINI scores (as an assessment of degree of equality in countries) and age discrimination experienced among older and younger respondents.

Most of our analyses focused on the AgeWatch Index, as this index more specifically targets social and financial policies benefittig older people. Some of the indicators for the AgeWatch Index are similar to the indicators used by the HDI. Both include life expectancy, albeit at birth (the HDI) or at age 60 (the AgeWatch Index), but the AgeWatch Index also includes healthy life expectancy at 60 and psychological wellbeing reported by older people as further indicators of health status — one of the four dimensions used in the AgeWatch Index. GDP per capital is also used by both indices, but the AgeWatch Index adds three age-specific indicators to develop a measure of security of older people. Education is included in both indices, but the HDI uses an overall measure of education level, whereas the AgeWatch Index uses a measure specific for older people and adds employment of older people as a second indicator of the employment and education dimension. Finally, the AgeWatch Index adds a dimension not considered by the HDI, which is the extent to which environments are enabling for older people (with social connections, physical safety, civic freedom and access to public transport as indicators).

The two indices correlated strongly ($r = .89$ when the AgeWatch Index from 2013 is compared with the HDI from 2008, see Table S2, and $r = .90$ when the AgeWatch Index and the HDI from 2013 are compared). The very strong correlation was consistent with our hypothesis that financial and social support to older people will be a significant mediator of associations between modernization and experiences of age discrimination. Results in analyses of age discrimination were similar when the HDI was used as a country-level predictor instead of the AgeWatch Index (see later Figure S2 for analyses with the HDI).

Table S1: Frequences of the three age discrimination items, in percents

	Older respondents	Younger respondents
**Experiences of prejudice**		
0 - Never	68.36	52.96
1	15.18	19.18
2	8.95	14.94
3	5.11	9.82
4 - Very often	2.40	3.10
**Experiencs of lack of respect**		
0 - Never	65.03	48.89
1	16.83	22.35
2	9.99	15.37
3	5.69	10.32
4 - Very often	2.46	3.07
**Being treaded badly**		
0 - Never	71.92	61.53
1	15.18	20.94
2	7.67	10.89
3	3.78	5.18
4 - Very often	1.45	1.47

Descriptive Statistics

Table S1 shows frequencies at the individual level for the three age discrimination items. Table S2 focuses on the country level (with $n = 29$), showing correlations between country-level composite scores of experiences of age discrimination in both age groups and country-level predictors of discrimination (HDI, the AgeWatch Index, and social norms against age discrimination).

Table S2: Correlation table for country-level composite scores of age discrimination and predictors ($N = 29$, p-values in parentheses)

		1	2	3	4	5
1	Age discrimination experienced by older respondents					
2	Age discrimination experienced by younger respondents	-.181				
		(.348)				
3	Human Development Index	-.658	.507			
		(.000)	(.005)			
4	AgeWatch Index	-.715	.552	.890		
		(.000)	(.002)	(.000)		
5	Social norm among respondents younger than 70	-.712	.133	.638	.576	
		(.000)	(.491)	(.000)	(.001)	
6	Social norm among respondents 30 years or older	-.689	.100	.630	.555	.992
		(.000)	(.607)	(.000)	(.002)	(.000)

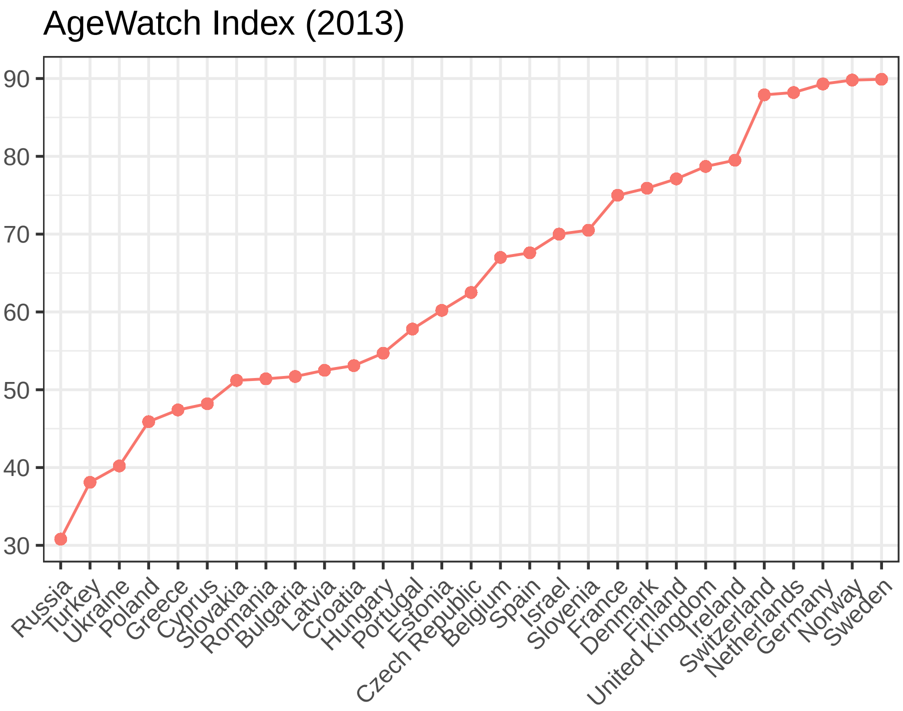

Figure S1: Countries' scores on the AgeWatch Index

The data had very few missing responses (see Table S3). Ninety seven percent among older respondents and 98% among younger respondents responded to all ESS items included in individual-level analyses. Analyses used full information methods, i.e., the included individuals who had partially missing data. In total 8,122 respondents 70 years or older participated in the ESS, of which $8,117$ could be used for analyses by having responded to at least one of the three age discrimination items. In total, 11,650 respondents below 30 years participated in the ESS, of which $11,647$ could be used for analyses.

Table S3: Patterns of missing data (x = not missing)

	1	2	3	4	5	6	7	8	9	10
Prejudice	x	x	x	x	x	x				
Lack of respect	x	x	x	x			x	x		
Treated badly	x	x			x		x		x	
Gender	x		x		x	x	x	x	x	x
Older respondents, percents	96.65	0.05	0.43	0.01	0.47	0.36	0.96	0.05	0.21	0.81
Younger respondents, percents	97.87	0.03	0.35	0.00	0.37	0.27	0.48	0.07	0.09	0.47

Table note. The first three items in the table refer to experiences of age discrimination (i.e., prejudice, lack of respect, and treated badly because of age).

Countries' scores on the AgeWatch Index are shown in Figure S1 and Table S4.

Table S4: Countries' scores on the AgeWatch Index

Country	AgeWatch Index
Russia	30.8
Turkey	38.1
Ukraine	40.2
Poland	45.9
Greece	47.4
Cyprus	48.2
Slovakia	51.2
Romania	51.4
Bulgaria	51.7
Latvia	52.5
Croatia	53.1
Hungary	54.7
Portugal	57.8
Estonia	60.2
Czech Republic	62.5
Belgium	67.0
Spain	67.6
Israel	70.0
Slovenia	70.5
France	75.0
Denmark	75.9
Finland	77.1
United Kingdom	78.7
Ireland	79.5
Switzerland	87.9
Netherlands	88.2
Germany	89.3
Norway	89.8
Sweden	89.9

Plots using the Human Development Index and the GINI Index

Figure S2 shows how age discrimination experienced among older and younger respondents was associated with (a) the HDI and (b) the GINI. The HDI scores are from 2008, the GINI scores are mostly from 2008. For Germany and Isreal, we used the GINI scores from 2007 since scores for 2008 were unavailable. For Croatia, we used the 2009 score since neither 2008 nor 2007 scores were available. GINI data was not available for Poland. GINI scores were recoded into a scale from that had 0 and 17.9 as extreme values (new scores = 41.6 - GINI). The figure uses two-letter ISO codes for countries, explained below in Table S5.

Table S5: ISO codes and country names

ISO code	Country
BE	Belgium
BG	Bulgaria
CH	Switzerland
CY	Cyprus
CZ	Check Republic
DE	Germany
DK	Denmark
EE	Estonia
ES	Spain
FI	Finland
FR	France
GB	United Kingdom
GR	Greece
HR	Croatia
HU	Hungary
IE	Ireland
IL	Israel
LV	Latvia
NL	Netherlands
NO	Norway
PL	Polen
PT	Portugal
RO	Romania
RU	Russia
SE	Sweden
SI	Slovenia
SK	Slovakia
TR	Turkey
UA	Ukraine

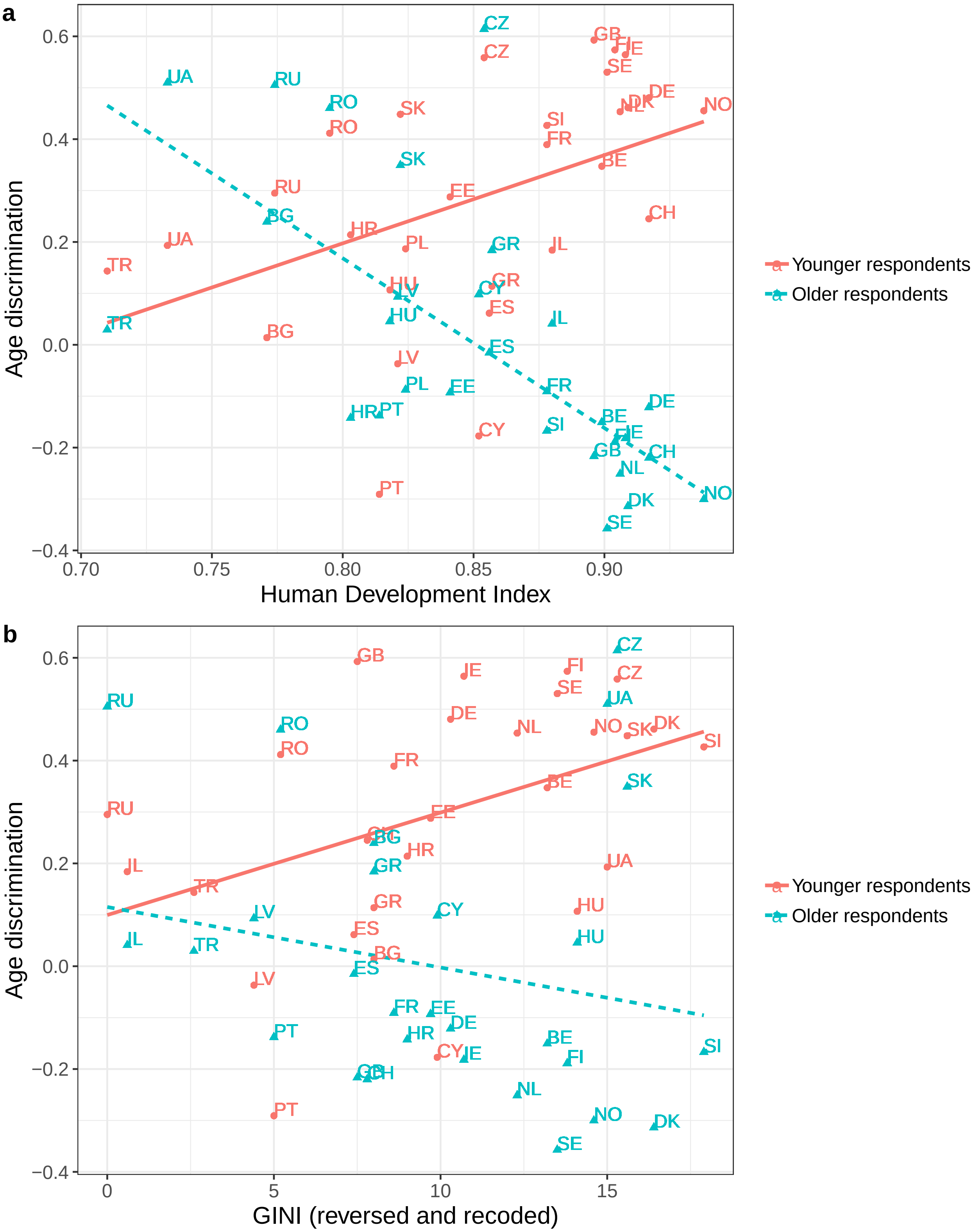

Figure S2: Comparisons of country-level scores for age discrimination experienced among younger and older respondents and (a) the HDI or (b) the GINI.

Plots using AgeWatch Index Scores and Social Norms

Figure S3 on the next page repeats plots from Figure 1 in the article, but adds two-letter ISO codes for countries. We also include plots where potential outliers have been dropped (Figure S4).

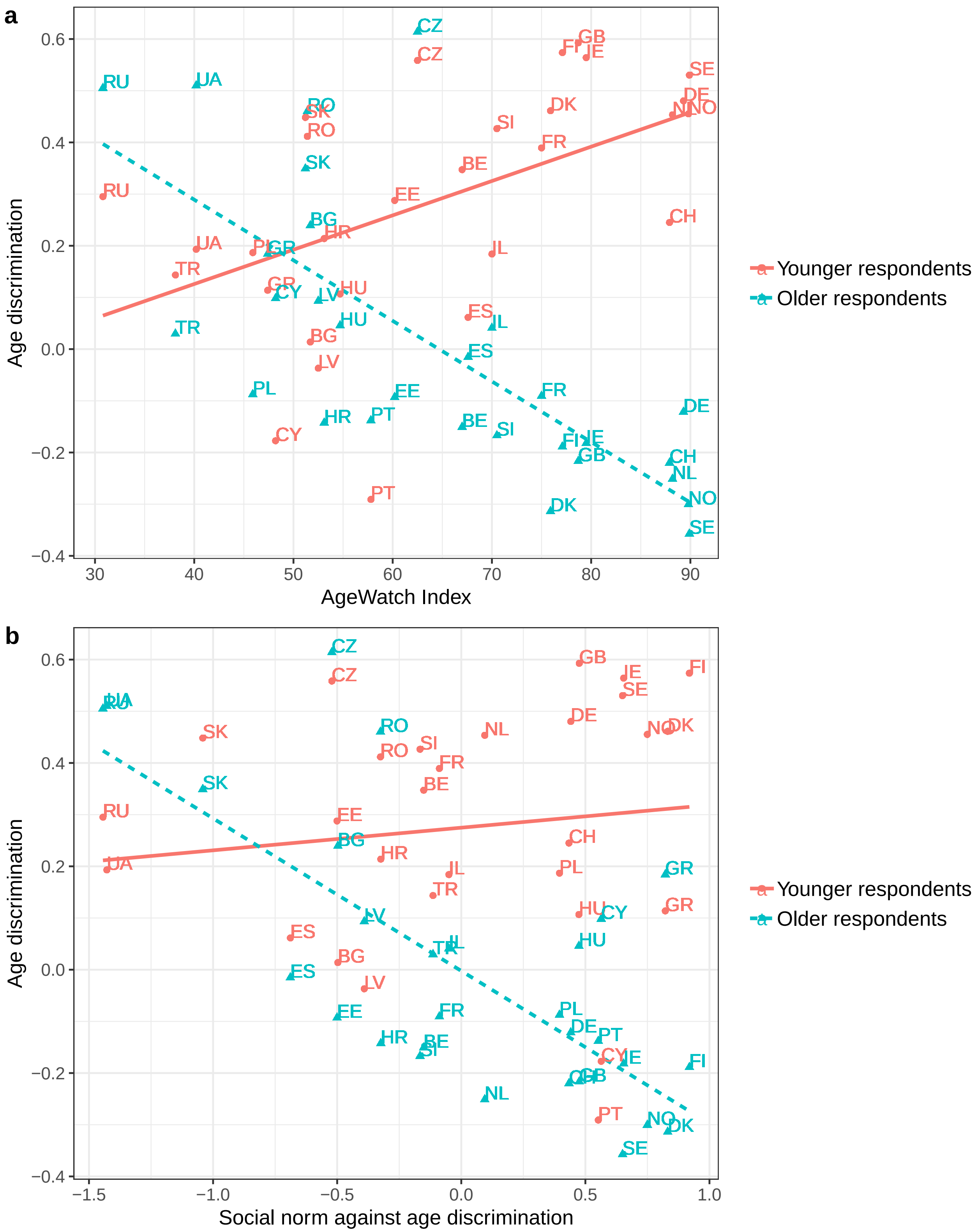

Figure S3: Country differences in age discrimination experienced by older and younger respondents, dependent on AgeWatch Index scores and social norms against age discrimination.

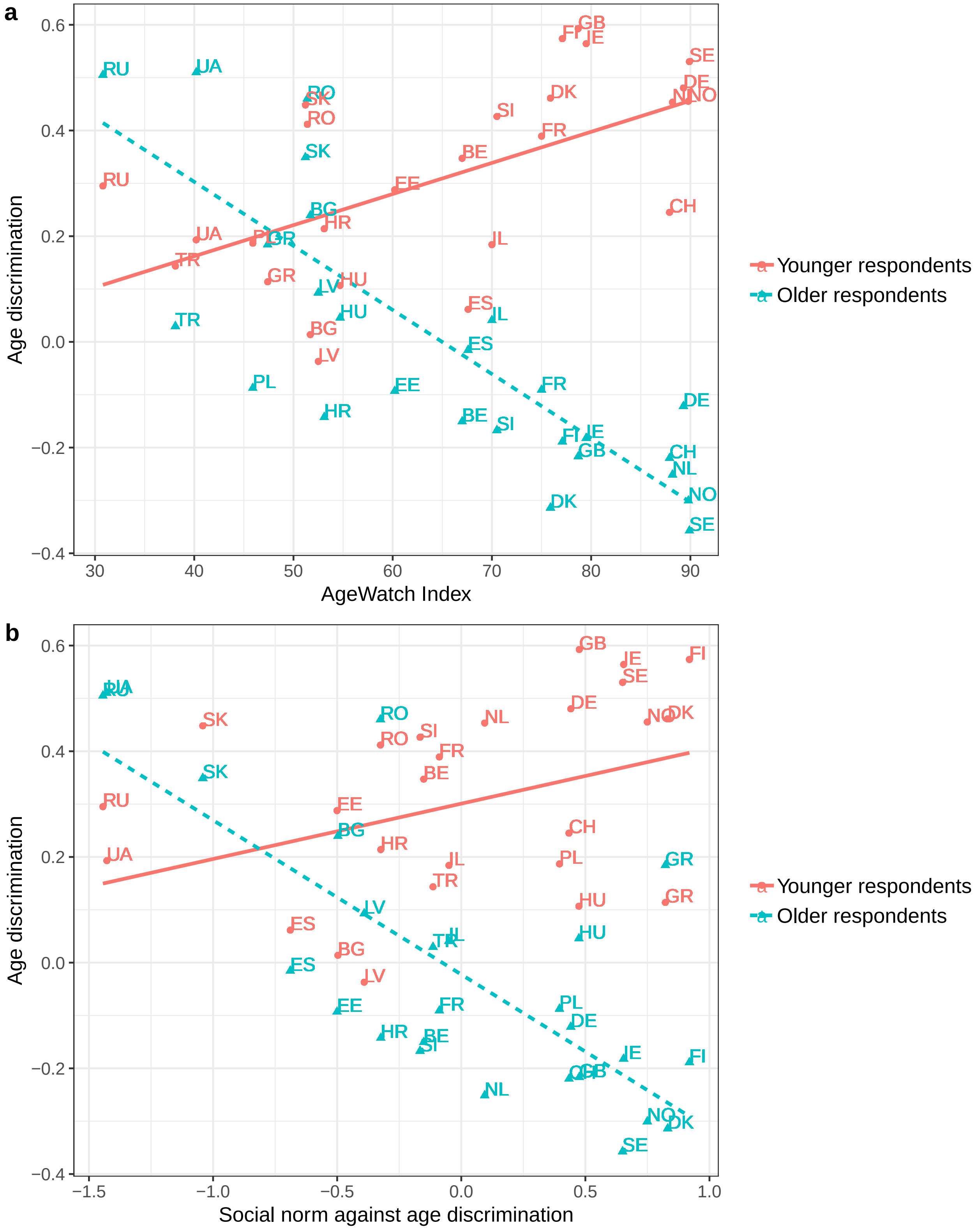

Figure S4: Country differences in age discrimination experienced by older and younger respondents, dependent on AgeWatch Index scores and social norms against age discrimination. Portugal, Cyprus, and Czechia dropped.

Age Watch Index

**Social norms**	High	Low
Strong	$M_o = 0.34$ $M_y = 1.06$<br>CH, DK, DE, FI, GB, IE, NL, NO, SE	$M_o = 0.53$ $M_y = 0.57$<br>CY, GR, HU, PL, PT
Weak	$M_o = 0.50$ $M_y = 0.81$<br>BE, ES, FR, IL, SI	$M_o = 0.84$ $M_y = 0.81$<br>BG, CZ, EE, HR, LV, RO, RU, SK, TR, UA

Table note. M_o refers to mean score for perceived age discrimination among older respondents, M_y refers to mean score for perceived age discrimination among younger respondents. Countries are listed using two-letter ISO codes.

Figure S5: Comparison of four groups of countries, dependent on scores on the AgeWatch Index (high or low) and social norms (strong or weak)

Comparing Four Groups of Countries

A reviewer suggested that we should compare groups of countries, based on their scores on the AgeWatch Index and social norms against age discrimination. Figure S5 shows a table distinguishing between four groups of countries, dependent on whether scores were high (above the mean) or low on the Age Watch Index and whether social norms against age discrimination were strong (above the mean) or weak. For each group of countries, the table shows the mean of composite scores for experiences of age discrimination, distinguishing between older respondents (M_o) and younger respondents (M_y). The table uses two-letter ISO codes to name countries, see Table S5 for an explanation. We included tests with ANOVA (using Bonferri tests of pairwise comparisons in multiple group comparisons).

Countries with high scores on the AgeWatch Index and strong social norms against age discrimination (seemingly an ideal combination for counteracting age discrimination) had the lowest reported frequency of age discrimination against older respondents, but the highest against younger respondents (mean score = 0.34 vs. 1.06, $p < .001$). Experiences of age discrimination were also more frequent among younger respondents than among older respondents in countries with high scores on the AgeWatch Index but weak social norms (0.50 vs 0.81, $p < .001$). However, no differences between older and younger respondents were evident in countries with *low* scores on the AgeWatch Index (independently of whether the social norm was strong, $M = 0.53$ vs. 0.57, $p = .231$, or if the social norm was weak, $M = 0.84$ vs. 0.81, $p = .227$).

Wheras older respondents reported the least age discrimination in countries that combined high scores on the AgeWatch Index and strong social norms, they reported the most frequent age discrimination in countries with low scores on the AgeWatch Index and weak social norms against age discrimination. No difference in older respondents' resports was evident between countries with either high AgeWatch Index scores and weak social norms ($M = 0.50$) or low AgeWatch Index scores and strong social norms ($M = 0.53$, $p = 1.00$).

Younger respondents experienced the least age discrimination in countries with low scores on the AgeWatch Index and strong social norms against age discrimination. No difference was evident when comparing countries that had weak social norms and either high ($M = 0.81$) or low ($M = 0.81$) scores on the AgeWatch Index ($p = 1.00$).

We refer to Model 4 in multilevel SEM analyses for tests of interaction effects with continuous measurements. Those tests using continuous variables avoid using arbitary cutoffs to dichotomize variables.

Multilevel SEM: Maximum Likelihood Estimations

Tables S6 to S10 show results of multilevel analyses of experiences of age discrimination, using maximum likelihood estimations with robust standard errors (MLR). Table S6 uses HDI scores as the country-level predictor, Tables S7 to S10 focus on AgeWatch Index and social norms as country-level predictors.

Table S6: The Human Development Index as country-level predictor of experiences of age discrimination, maximum likelihood estimations (separate results for older and younger respondents)

	Older respondents		Younger respondents	
	Estimate	95% CI	Estimate	95% CI
**Individual level**				
Factor loadings				
Loading Prejudice	1.00	[1.00, 1.00]	1.00	[1.00, 1.00]
Loading Respect	1.78	[1.44, 2.12]	2.01	[1.65, 2.38]
Loading Treated	1.79	[1.46, 2.13]	1.45	[1.22, 1.68]
Regression weights				
Female	0.27	[0.05, 0.49]	0.12	[0.01, 0.24]
Age	0.01	[-0.01, 0.03]	-0.07	[-0.09, -0.05]
Explained variance				
Residual Discrimination	7.98	[6.18, 9.78]	5.41	[4.49, 6.33]
R-square	0.00		0.02	
**Country level**				
Factor loadings				
Loading Prejudice	1.00	[1.00, 1.00]	1.00	[1.00, 1.00]
Loading Respect	1.85	[1.32, 2.39]	1.40	[1.04, 1.77]
Loading Treated	2.10	[1.62, 2.58]	0.44	[0.13, 0.75]
Regression weight				
Human Development Index	-0.11	[-0.17, -0.05]	0.12	[0.08, 0.16]
Thresholds				
Threshold 1 Prejudice	-7.67	[-12.67, -2.67]	10.33	[6.73, 13.94]
Threshold 2 Prejudice	-5.93	[-10.89, -0.97]	11.90	[8.25, 15.55]
Threshold 3 Prejudice	-4.37	[-9.30, 0.57]	13.48	[9.80, 17.17]
Threshold 4 Prejudice	-2.46	[-7.39, 2.48]	15.65	[11.84, 19.46]
Threshold 1 Respect	-15.10	[-23.76, -6.44]	14.17	[9.08, 19.25]
Threshold 2 Respect	-12.02	[-20.48, -3.56]	17.25	[12.07, 22.42]
Threshold 3 Respect	-9.39	[-17.66, -1.11]	19.89	[14.68, 25.11]
Threshold 4 Respect	-6.33	[-14.49, 1.82]	23.62	[18.20, 29.05]
Threshold 1 Treated	-16.36	[-26.00, -6.72]	5.70	[1.95, 9.45]
Threshold 2 Treated	-13.22	[-22.81, -3.62]	8.07	[4.34, 11.81]
Threshold 3 Treated	-10.46	[-20.07, -0.85]	10.18	[6.47, 13.89]
Threshold 4 Treated	-7.37	[-17.00, 2.26]	12.84	[9.16, 16.52]
Explained variance				
Residual Discrimination	0.47	[0.13, 0.81]	0.58	[0.06, 1.11]
R-square	0.47		0.44	
**Model fit**				
Log likelihood	-17926.22		-34730.00	
Correction factor	3.18		5.44	
AIC	35894.44		69502.01	
BIC	36041.31		69656.53	

Note:
Estimates are unstandardized. The parameters with no compatibility interval (CI 1,00 to 1,00) were fixed prior to estimations. CI = compatibility interval (also called confidence interval), AIC = Akaike Information Criterion, BIC = Bayesian Information Criterion.

Table S7: Model 1. The AgeWatch Index as country-level predictor of experiences of age discrimination, maximum likelihood estimations (separate results for older and younger respondents)

	Older respondents		Younger respondents	
	Estimate	95% CI	Estimate	95% CI
**Individual level**				
Factor loadings				
Loading Prejudice	1.00	[1.00, 1.00]	1.00	[1.00, 1.00]
Loading Respect	1.78	[1.44, 2.12]	2.01	[1.65, 2.38]
Loading Treated	1.79	[1.46, 2.13]	1.45	[1.22, 1.68]
Regression weights				
Female	0.27	[0.05, 0.49]	0.12	[0.01, 0.24]
Age	0.01	[-0.01, 0.03]	-0.07	[-0.09, -0.05]
Explained variance				
Residual Discrimination	7.98	[6.19, 9.78]	5.41	[4.48, 6.34]
R-square	0.00		0.02	
**Country level**				
Factor loadings				
Loading Prejudice	1.00	[1.00, 1.00]	1.00	[1.00, 1.00]
Loading Respect	1.85	[1.32, 2.39]	1.40	[1.04, 1.77]
Loading Treated	2.11	[1.63, 2.58]	0.44	[0.13, 0.75]
Regression weight				
AgeWatch Index	-0.22	[-0.29, -0.15]	0.21	[0.13, 0.29]
Thresholds				
Threshold 1 Prejudice	1.88	[1.47, 2.28]	0.31	[-0.01, 0.62]
Threshold 2 Prejudice	3.61	[3.17, 4.06]	1.87	[1.58, 2.16]
Threshold 3 Prejudice	5.18	[4.64, 5.72]	3.46	[3.13, 3.79]
Threshold 4 Prejudice	7.09	[6.44, 7.75]	5.62	[5.16, 6.08]
Threshold 1 Respect	2.59	[1.72, 3.46]	0.10	[-0.40, 0.60]
Threshold 2 Respect	5.67	[4.33, 7.00]	3.18	[2.47, 3.89]
Threshold 3 Respect	8.30	[6.49, 10.11]	5.84	[4.82, 6.86]
Threshold 4 Respect	11.36	[8.91, 13.81]	9.57	[8.06, 11.08]
Threshold 1 Treated	3.70	[2.81, 4.59]	1.27	[0.97, 1.56]
Threshold 2 Treated	6.85	[5.70, 8.00]	3.64	[3.30, 3.98]
Threshold 3 Treated	9.61	[8.22, 10.99]	5.75	[5.30, 6.19]
Threshold 4 Treated	12.69	[10.88, 14.51]	8.40	[7.67, 9.14]
Explained variance				
Residual Discrimination	0.36	[0.09, 0.64]	0.50	[0.01, 0.98]
R-square	0.60		0.50	
**Model fit**				
Log likelihood	-17922.71		-34728.46	
Correction factor	3.16		5.48	
AIC	35887.42		69498.93	
BIC	36034.29		69653.45	

Note:
Estimates are unstandardized. The parameters with no compatibility interval (CI 1,00 to 1,00) were fixed prior to estimations. CI = compatibility interval (also called confidence interval), AIC = Akaike Information Criterion, BIC = Bayesian Information Criterion.

Table S8: Model 2. Social norms as country-level predictor of experiences of age discrimination, maximum likelihood estimations (separate results for older and younger respondents)

	Older respondents		Younger respondents	
	Estimate	95% CI	Estimate	95% CI
**Individual level**				
Factor loadings				
Loading Prejudice	1.00	[1.00, 1.00]	1.00	[1.00, 1.00]
Loading Respect	1.78	[1.44, 2.12]	2.01	[1.65, 2.38]
Loading Treated	1.79	[1.46, 2.13]	1.45	[1.22, 1.68]
Regression weights				
Female	0.27	[0.05, 0.49]	0.12	[0.01, 0.24]
Age	0.00	[-0.01, 0.03]	-0.07	[-0.09, -0.05]
Explained variance				
Residual Discrimination	7.96	[6.17, 9.76]	5.40	[4.47, 6.33]
R-square	0.00		0.02	
**Country level**				
Factor loadings				
Loading Prejudice	1.00	[1.00, 1.00]	1.00	[1.00, 1.00]
Loading Respect	1.84	[1.32, 2.37]	1.41	[1.04, 1.77]
Loading Treated	2.08	[1.61, 2.55]	0.45	[0.14, 0.75]
Regression weight				
Social norm	-1.04	[-1.45, -0.63]	0.24	[-0.29, 0.78]
Thresholds				
Threshold 1 Prejudice	1.85	[1.48, 2.21]	0.31	[-0.09, 0.71]
Threshold 2 Prejudice	3.59	[3.18, 4.00]	1.88	[1.51, 2.24]
Threshold 3 Prejudice	5.16	[4.65, 5.66]	3.46	[3.06, 3.86]
Threshold 4 Prejudice	7.07	[6.45, 7.68]	5.63	[5.16, 6.10]
Threshold 1 Respect	2.54	[1.76, 3.33]	0.11	[-0.48, 0.70]
Threshold 2 Respect	5.62	[4.40, 6.84]	3.19	[2.33, 4.05]
Threshold 3 Respect	8.26	[6.56, 9.96]	5.84	[4.65, 7.04]
Threshold 4 Respect	11.32	[8.99, 13.64]	9.57	[7.89, 11.26]
Threshold 1 Treated	3.65	[2.72, 4.57]	1.27	[0.97, 1.57]
Threshold 2 Treated	6.79	[5.62, 7.96]	3.64	[3.28, 4.00]
Threshold 3 Treated	9.55	[8.15, 10.94]	5.75	[5.27, 6.22]
Threshold 4 Treated	12.63	[10.79, 14.48]	8.40	[7.64, 9.16]
Explained variance				
Residual Discrimination	0.46	[0.16, 0.74]	1.01	[0.28, 1.75]
R-square	0.51		0.02	
**Model fit**				
Log likelihood	-17925.10		-34737.63	
Correction factor	3.15		5.43	
AIC	35892.20		69517.26	
BIC	36039.07		69671.78	

Note:
Estimates are unstandardized. The parameters with no compatibility interval (CI 1,00 to 1,00) were fixed prior to estimations. CI = compatibility interval (also called confidence interval), AIC = Akaike Information Criterion, BIC = Bayesian Information Criterion.

Table S9: Model 3. The AgeWatch Index and social norms as country-level predictors of experiences of age discrimination, maximum likelihood estimations (separate results for older and younger respondents)

	Older respondents		Younger respondents	
	Estimate	95% CI	Estimate	95% CI
**Individual level**				
Factor loadings				
Loading Prejudice	1.00	[1.00, 1.00]	1.00	[1.00, 1.00]
Loading Respect	1.78	[1.44, 2.12]	2.01	[1.65, 2.38]
Loading Treated	1.79	[1.46, 2.13]	1.45	[1.22, 1.68]
Regression weights				
Female	0.27	[0.05, 0.49]	0.12	[0.01, 0.24]
Age	0.01	[-0.01, 0.03]	-0.07	[-0.09, -0.05]
Explained variance				
Residual Discrimination	7.97	[6.18, 9.76]	5.41	[4.48, 6.33]
R-square	0.00		0.02	
**Country level**				
Factor loadings				
Loading Prejudice	1.00	[1.00, 1.00]	1.00	[1.00, 1.00]
Loading Respect	1.84	[1.31, 2.38]	1.40	[1.04, 1.77]
Loading Treated	2.10	[1.62, 2.57]	0.44	[0.13, 0.75]
Regression weights				
AgeWatch Index	-0.16	[-0.23, -0.09]	0.27	[0.17, 0.36]
Social Norms	-0.59	[-1.01, -0.16]	-0.47	[-1.01, 0.07]
Thresholds				
Threshold 1 Prejudice	1.86	[1.48, 2.23]	0.30	[0.01, 0.60]
Threshold 2 Prejudice	3.60	[3.16, 4.03]	1.87	[1.58, 2.15]
Threshold 3 Prejudice	5.16	[4.63, 5.69]	3.46	[3.14, 3.77]
Threshold 4 Prejudice	7.07	[6.42, 7.72]	5.62	[5.17, 6.07]
Threshold 1 Respect	2.56	[1.72, 3.39]	0.10	[-0.39, 0.58]
Threshold 2 Respect	5.64	[4.32, 6.96]	3.18	[2.48, 3.88]
Threshold 3 Respect	8.28	[6.46, 10.09]	5.83	[4.83, 6.84]
Threshold 4 Respect	11.33	[8.89, 13.78]	9.57	[8.08, 11.06]
Threshold 1 Treated	3.66	[2.82, 4.50]	1.27	[0.98, 1.56]
Threshold 2 Treated	6.81	[5.70, 7.92]	3.64	[3.30, 3.98]
Threshold 3 Treated	9.56	[8.22, 10.91]	5.75	[5.31, 6.19]
Threshold 4 Treated	12.65	[10.87, 14.43]	8.40	[7.68, 9.13]
Explained variance				
Residual Discrimination	0.26	[0.06, 0.47]	0.46	[0.03, 0.90]
R-square	0.72		0.56	
**Model fit**				
Log likelihood	-17918.42		-34726.49	
Correction factor	3.07		5.26	
AIC	35880.85		69496.99	
BIC	36034.71		69658.86	

Note:
Estimates are unstandardized. The parameters with no compatibility interval (CI 1,00 to 1,00) were fixed prior to estimations. CI = compatibility interval (also called confidence interval), AIC = Akaike Information Criterion, BIC = Bayesian Information Criterion.

Table S10: Model 4. The AgeWatch Index, social norms, and their interaction as country-level predictors of experiences of age discrimination, maximum likelihood estimations (separate results for older and younger respondents)

	Older respondents		Younger respondents	
	Estimate	95% CI	Estimate	95% CI
**Individual level**				
Factor loadings				
Loading Prejudice	1.00	[1.00, 1.00]	1.00	[1.00, 1.00]
Loading Respect	1.78	[1.44, 2.13]	2.01	[1.65, 2.38]
Loading Treated	1.79	[1.46, 2.13]	1.45	[1.22, 1.68]
Regression weights				
Female	0.27	[0.05, 0.49]	0.12	[0.01, 0.24]
Age	0.00	[-0.01, 0.03]	-0.07	[-0.09, -0.05]
Explained variance				
Residual Discrimination	7.97	[6.18, 9.76]	5.41	[4.48, 6.33]
R-square	0.00		0.02	
**Country level**				
Factor loadings				
Loading Prejudice	1.00	[1.00, 1.00]	1.00	[1.00, 1.00]
Loading Respect	1.85	[1.31, 2.38]	1.40	[1.04, 1.77]
Loading Treated	2.10	[1.62, 2.57]	0.44	[0.14, 0.75]
Regression weights				
AgeWatch Index	-0.15	[-0.22, -0.09]	0.24	[0.15, 0.32]
Social Norms	-0.58	[-1.07, -0.08]	-0.21	[-0.69, 0.27]
Interaction	-0.01	[-0.09, 0.07]	0.14	[0.05, 0.23]
Thresholds				
Threshold 1 Prejudice	1.85	[1.45, 2.25]	0.48	[0.14, 0.81]
Threshold 2 Prejudice	3.59	[3.14, 4.03]	2.04	[1.70, 2.39]
Threshold 3 Prejudice	5.15	[4.62, 5.68]	3.63	[3.24, 4.02]
Threshold 4 Prejudice	7.06	[6.41, 7.71]	5.80	[5.28, 6.31]
Threshold 1 Respect	2.54	[1.64, 3.44]	0.35	[-0.22, 0.91]
Threshold 2 Respect	5.62	[4.28, 6.96]	3.43	[2.65, 4.21]
Threshold 3 Respect	8.26	[6.45, 10.07]	6.08	[4.99, 7.17]
Threshold 4 Respect	11.32	[8.88, 13.76]	9.81	[8.26, 11.37]
Threshold 1 Treated	3.64	[2.70, 4.58]	1.35	[1.02, 1.67]
Threshold 2 Treated	6.78	[5.60, 7.97]	3.72	[3.37, 4.07]
Threshold 3 Treated	9.54	[8.14, 10.94]	5.83	[5.39, 6.26]
Threshold 4 Treated	12.63	[10.79, 14.46]	8.49	[7.78, 9.19]
Explained variance				
Residual Discrimination	0.27	[0.06, 0.47]	0.36	[0.01, 0.71]
R-square	0.70		0.66	
**Model fit**				
Log likelihood	-17918.44		-34723.17	
Correction factor	2.96		5.03	
AIC	35882.89		69492.34	
BIC	36043.74		69661.57	

Note:
Estimates are unstandardized. The parameters with no compatibility interval (CI 1,00 to 1,00) were fixed prior to estimations. CI = compatibility interval (also called confidence interval), AIC = Akaike Information Criterion, BIC = Bayesian Information Criterion.

Multilevel SEM: Bayesian Estimations

All multilevel SEM Models 1 to 4 were also estimated with Bayesian estimations, the results are shown in Tables S11 to S14 below. The country-level regression weights for both MLR and Bayesian estimations are summarized in Figure S6, easing the comparison of parameters across estimation methods. Overall, the Bayesian estimations supported the findings in the earlier estimations with MLR, though country-level regression weights tended to be lower with Bayesian estimations (reflecting the more conservative nature of these estimations). The 95% credible intervals in Bayesian estimations were narrower than the 95% confidence intervals in MLR estimations. The Bayesian credible interval for the interaction effect between AgeWatch Index and social norms (Model 4) included both negative and positive values [CI = -0.02, 0.14].

The Posterior Predictive P-Value (PPP) indicated very good fit for all analyses of older respondents (PPP between .34 and .41). The sample of younger respondents was larger and resulted in the PPP below .05 in models that included the AgeWatch Index as a predictor. The tables also show the Potential Scale Reduction (PSR) for the four Markoc chain Monte Carlo (MCMC) used, with separate values for the last iteration in the burn-in (after half of the total number of iterations) and in the last iteration of the overall analysis. Due to the many Bayesian iterations used, the PSR values were consistently 1.00 or 1.01 already after the initial burn-in of the two MCMC chains.

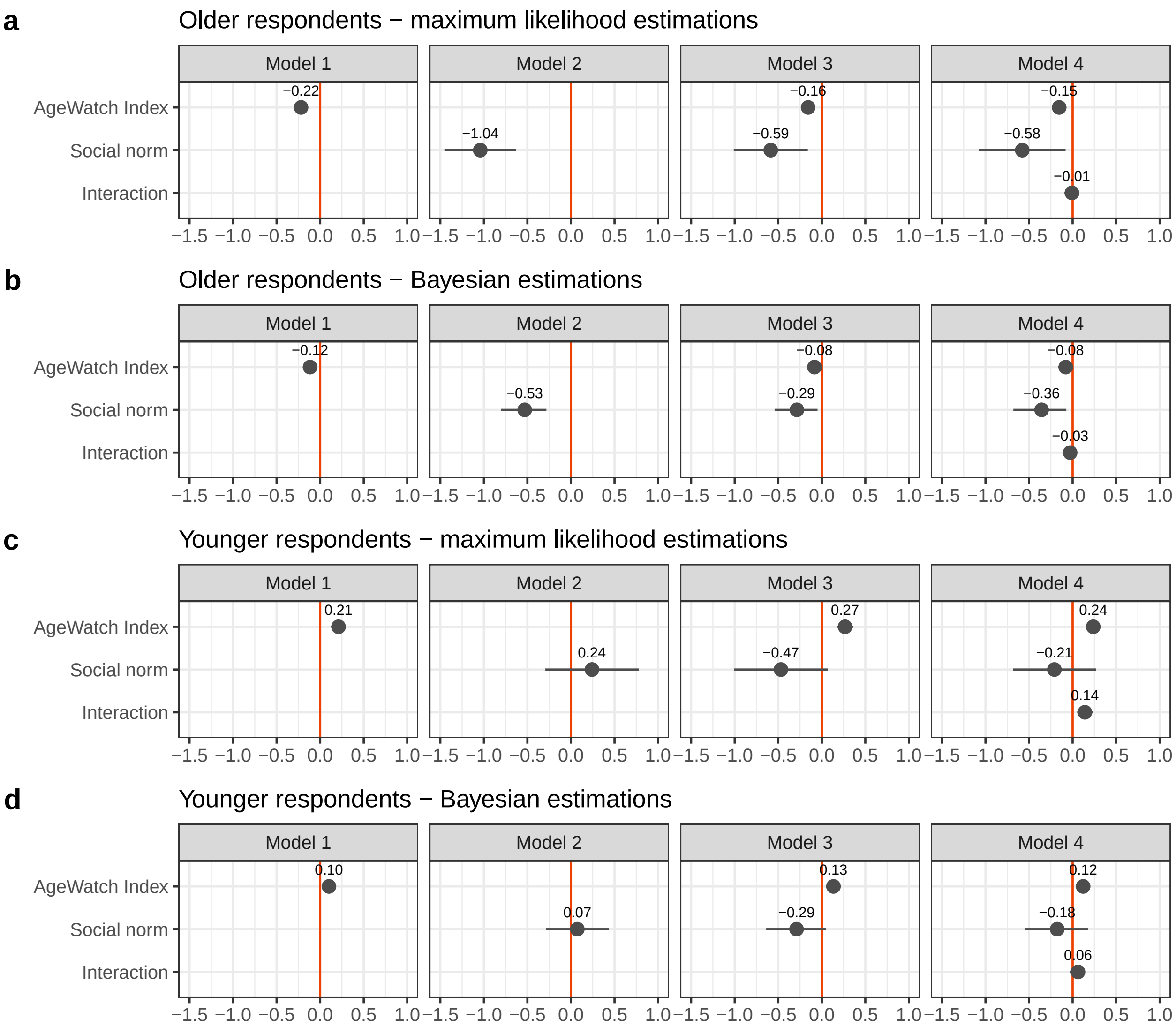

Figure S6: The AgeWatch Index and social norms against age discrimination predicting experiences of age discrimination: (a) Older respondents, MLR estimations, (b) Older respondents, Bayesian estimations, (c) Younger resondents, MLR estimations, (d) Younger respondents, Bayesian estimations

Table S11: Model 1 (Bayesian estimations). The AgeWatch Index as country-level predictor of experiences of age discrimination (separate results for older and younger respondents)

	Older respondents		Younger respondents	
	Estimate	95% CI	Estimate	95% CI
**Individual level**				
Factor loadings				
Loading Prejudice	1.00	[1.00, 1.00]	1.00	[1.00, 1.00]
Loading Respect	1.91	[1.73, 2.13]	2.09	[1.93, 2.30]
Loading Treated	1.62	[1.50, 1.76]	1.37	[1.30, 1.44]
Regression weights				
Female	0.11	[0.02, 0.19]	0.07	[0.02, 0.13]
Age	0.00	[-0.01, 0.01]	-0.04	[-0.05, -0.03]
Explained variance				
Residual Discrimination	2.45	[2.21, 2.71]	1.77	[1.64, 1.91]
R-square	0.00	[0.00, 0.00]	0.02	[0.01, 0.02]
**Country level**				
Factor loadings				
Loading Prejudice	1.00	[1.00, 1.00]	1.00	[1.00, 1.00]
Loading Respect	1.99	[1.57, 2.53]	1.58	[1.23, 2.01]
Loading Treated	1.96	[1.61, 2.43]	0.64	[0.39, 0.93]
Regression weight				
AgeWatch Index	-0.12	[-0.17, -0.07]	0.10	[0.04, 0.16]
Thresholds				
Threshold 1 Prejudice	0.94	[0.75, 1.11]	0.14	[-0.09, 0.35]
Threshold 2 Prejudice	1.90	[1.70, 2.08]	1.04	[0.81, 1.25]
Threshold 3 Prejudice	2.76	[2.55, 2.95]	1.94	[1.71, 2.16]
Threshold 4 Prejudice	3.73	[3.50, 3.94]	3.11	[2.88, 3.34]
Threshold 1 Respect	1.30	[0.95, 1.62]	-0.04	[-0.40, 0.29]
Threshold 2 Respect	3.06	[2.66, 3.45]	1.81	[1.43, 2.17]
Threshold 3 Respect	4.58	[4.12, 5.06]	3.38	[2.97, 3.80]
Threshold 4 Respect	6.25	[5.71, 6.84]	5.47	[4.99, 5.99]
Threshold 1 Treated	1.68	[1.35, 1.98]	0.69	[0.49, 0.88]
Threshold 2 Treated	3.23	[2.87, 3.57]	2.00	[1.80, 2.20]
Threshold 3 Treated	4.58	[4.18, 4.97]	3.15	[2.94, 3.37]
Threshold 4 Treated	6.08	[5.62, 6.55]	4.53	[4.28, 4.78]
Explained variance				
Residual Discrimination	0.12	[0.06, 0.27]	0.24	[0.13, 0.49]
R-square	0.53	[0.24, 0.72]	0.30	[0.06, 0.55]
**Model fit**				
PSR, after burn-in	1.00		1.00	
PSR, end of iterations	1.00		1.00	
Posterior Predictive P-Value	0.30		0.01	

Note:
Estimates are unstandardized. Parameters with no credibility interval [1.00, 1.00] were fixed prior to estimations. CI = Credibility interval, PSR = Potential Scale Reduction.

Table S12: Model 2 (Bayesian estimations). Social norms as country-level predictor of experiences of age discrimination (separate results for older and younger respondents)

	Older respondents		Younger respondents	
	Estimate	95% CI	Estimate	95% CI
**Individual level**				
Factor loadings				
Loading Prejudice	1.00	[1.00, 1.00]	1.00	[1.00, 1.00]
Loading Respect	1.91	[1.74, 2.13]	2.09	[1.93, 2.30]
Loading Treated	1.62	[1.50, 1.76]	1.37	[1.30, 1.44]
Regression weights				
Female	0.10	[0.02, 0.19]	0.07	[0.02, 0.13]
Age	0.00	[-0.01, 0.01]	-0.04	[-0.05, -0.03]
Explained variance				
Residual Discrimination	2.45	[2.21, 2.71]	1.77	[1.64, 1.91]
R-square	0.00	[0.00, 0.00]	0.02	[0.01, 0.02]
**Country level**				
Factor loadings				
Loading Prejudice	1.00	[1.00, 1.00]	1.00	[1.00, 1.00]
Loading Respect	1.97	[1.57, 2.50]	1.62	[1.28, 2.07]
Loading Treated	1.91	[1.57, 2.37]	0.68	[0.43, 0.97]
Regression weight				
Social norm	-0.53	[-0.80, -0.28]	0.07	[-0.29, 0.43]
Thresholds				
Threshold 1 Prejudice	0.92	[0.72, 1.11]	0.11	[-0.19, 0.37]
Threshold 2 Prejudice	1.87	[1.66, 2.06]	1.01	[0.71, 1.27]
Threshold 3 Prejudice	2.74	[2.51, 2.94]	1.91	[1.61, 2.18]
Threshold 4 Prejudice	3.70	[3.47, 3.93]	3.09	[2.78, 3.36]
Threshold 1 Respect	1.26	[0.88, 1.62]	-0.08	[-0.55, 0.31]
Threshold 2 Respect	3.02	[2.60, 3.44]	1.77	[1.28, 2.19]
Threshold 3 Respect	4.54	[4.07, 5.05]	3.34	[2.83, 3.80]
Threshold 4 Respect	6.21	[5.65, 6.83]	5.42	[4.86, 5.98]
Threshold 1 Treated	1.64	[1.28, 1.96]	0.67	[0.44, 0.87]
Threshold 2 Treated	3.19	[2.80, 3.54]	1.98	[1.75, 2.19]
Threshold 3 Treated	4.54	[4.11, 4.94]	3.13	[2.89, 3.36]
Threshold 4 Treated	6.04	[5.55, 6.51]	4.51	[4.23, 4.77]
Explained variance				
Residual Discrimination	0.16	[0.08, 0.35]	0.37	[0.19, 0.76]
R-square	0.26	[0.07, 0.47]	0.02	[0.00, 0.17]
**Model fit**				
PSR, after burn-in	1.01		1.00	
PSR, end of iterations	1.00		1.00	
Posterior Predictive P-Value	0.38		0.06	

Note:
Estimates are unstandardized. Parameters with no credibility interval [1.00, 1.00] were fixed prior to estimations. CI = Credibility interval, PSR = Potential Scale Reduction.

Table S13: Model 3 (Bayesian estimations). The AgeWatch Index and social norms as country-level predictors of experiences of age discrimination (separate results for older and younger respondents)

	Older respondents		Younger respondents	
	Estimate	95% CI	Estimate	95% CI
**Individual level**				
Factor loadings				
Loading Prejudice	1.00	[1.00, 1.00]	1.00	[1.00, 1.00]
Loading Respect	1.91	[1.73, 2.12]	2.09	[1.92, 2.30]
Loading Treated	1.62	[1.50, 1.75]	1.36	[1.30, 1.44]
Regression weights				
Female	0.10	[0.02, 0.19]	0.07	[0.02, 0.13]
Age	0.00	[-0.01, 0.01]	-0.04	[-0.05, -0.03]
Explained variance				
Residual Discrimination	2.45	[2.22, 2.71]	1.77	[1.64, 1.91]
R-square	0.00	[0.00, 0.00]	0.02	[0.01, 0.02]
**Country level**				
Factor loadings				
Loading Prejudice	1.00	[1.00, 1.00]	1.00	[1.00, 1.00]
Loading Respect	1.96	[1.55, 2.48]	1.55	[1.21, 1.97]
Loading Treated	1.93	[1.59, 2.38]	0.63	[0.38, 0.91]
Regression weight				
AgeWatch Index	-0.08	[-0.14, -0.04]	0.13	[0.06, 0.21]
Social norm	-0.29	[-0.54, -0.05]	-0.29	[-0.64, 0.05]
Thresholds				
Threshold 1 Prejudice	0.94	[0.77, 1.11]	0.13	[-0.07, 0.32]
Threshold 2 Prejudice	1.90	[1.71, 2.07]	1.03	[0.83, 1.22]
Threshold 3 Prejudice	2.76	[2.57, 2.95]	1.93	[1.73, 2.13]
Threshold 4 Prejudice	3.73	[3.52, 3.94]	3.11	[2.90, 3.31]
Threshold 1 Respect	1.31	[0.96, 1.63]	-0.05	[-0.35, 0.24]
Threshold 2 Respect	3.06	[2.67, 3.46]	1.80	[1.48, 2.12]
Threshold 3 Respect	4.58	[4.13, 5.09]	3.37	[3.01, 3.75]
Threshold 4 Respect	6.24	[5.71, 6.87]	5.46	[5.01, 5.95]
Threshold 1 Treated	1.68	[1.38, 1.96]	0.68	[0.50, 0.86]
Threshold 2 Treated	3.23	[2.90, 3.55]	1.99	[1.81, 2.18]
Threshold 3 Treated	4.58	[4.22, 4.96]	3.14	[2.94, 3.35]
Threshold 4 Treated	6.08	[5.65, 6.53]	4.52	[4.28, 4.77]
Explained variance				
Residual Discrimination	0.10	[0.05, 0.23]	0.22	[0.12, 0.46]
R-square	0.53	[0.26, 0.72]	0.41	[0.13, 0.64]
**Model fit**				
PSR, after burn-in	1.00		1.05	
PSR, end of iterations	1.00		1.00	
PPP	0.29		0.01	

Note:
Estimates are unstandardized. Parameters with no credibility interval [1.00, 1.00] were fixed prior to estimations. CI = Credibility interval, PSR = Potential Scale Reduction.

Table S14: Model 4 (Bayesian estimations). The AgeWatch Index and social norms as well as their interaction as country-level predictors of experiences of age discrimination (separate results for older and younger respondents)

	Older respondents		Younger respondents	
	Estimate	95% CI	Estimate	95% CI
**Individual level**				
Factor loadings				
Loading Prejudice	1.00	[1.00, 1.00]	1.00	[1.00, 1.00]
Loading Respect	1.91	[1.74, 2.12]	2.10	[1.92, 2.30]
Loading Treated	1.62	[1.50, 1.75]	1.36	[1.30, 1.44]
Regression weights				
Female	0.10	[0.02, 0.19]	0.07	[0.02, 0.13]
Age	0.00	[-0.01, 0.01]	-0.04	[-0.05, -0.03]
Explained variance				
Residual Discrimination	2.45	[2.21, 2.71]	1.77	[1.64, 1.91]
R-square	0.00	[0.00, 0.00]	0.02	[0.01, 0.02]
**Country level**				
Factor loadings				
Loading Prejudice	1.00	[1.00, 1.00]	1.00	[1.00, 1.00]
Loading Respect	1.94	[1.54, 2.45]	1.54	[1.21, 1.95]
Loading Treated	1.92	[1.58, 2.35]	0.63	[0.38, 0.90]
Regression weight				
AgeWatch Index	-0.08	[-0.13, -0.03]	0.12	[0.05, 0.19]
Social norm	-0.36	[-0.68, -0.07]	-0.18	[-0.55, 0.18]
Interaction	-0.03	[-0.10, 0.03]	0.06	[-0.02, 0.14]
Thresholds				
Threshold 1 Prejudice	0.90	[0.70, 1.08]	0.21	[-0.04, 0.43]
Threshold 2 Prejudice	1.85	[1.64, 2.04]	1.11	[0.86, 1.33]
Threshold 3 Prejudice	2.72	[2.50, 2.91]	2.01	[1.76, 2.24]
Threshold 4 Prejudice	3.69	[3.45, 3.90]	3.19	[2.93, 3.42]
Threshold 1 Respect	1.22	[0.84, 1.56]	0.07	[-0.34, 0.41]
Threshold 2 Respect	2.97	[2.57, 3.38]	1.92	[1.50, 2.29]
Threshold 3 Respect	4.49	[4.04, 4.99]	3.49	[3.03, 3.91]
Threshold 4 Respect	6.16	[5.64, 6.76]	5.58	[5.05, 6.09]
Threshold 1 Treated	1.60	[1.24, 1.91]	0.73	[0.53, 0.93]
Threshold 2 Treated	3.15	[2.77, 3.49]	2.04	[1.83, 2.25]
Threshold 3 Treated	4.50	[4.09, 4.89]	3.19	[2.97, 3.42]
Threshold 4 Treated	6.00	[5.53, 6.46]	4.56	[4.31, 4.83]
Explained variance				
Residual Discrimination	0.11	[0.05, 0.25]	0.20	[0.10, 0.44]
R-square	0.56	[0.28, 0.75]	0.49	[0.19, 0.71]
**Model fit**				
PSR, after burn-in	1.00		1.00	
PSR, end of iterations	1.00		1.01	
PPP	0.26		0.02	

Note:
Estimates are unstandardized. Parameters with no credibility interval [1.00, 1.00] were fixed prior to estimations. CI = Credibility interval, PSR = Potential Scale Reduction.

Tests of Convergence of Bayesian Analyses

We tested convergence in the Bayesian analyses by inspecting Bayesian plots of parameters, and by doubling the number of Bayesian iterations and simultaneously using a different Bayesian seed number. Bayesian plots—trace plots, distribution plots, and autocorrelations plots—were inspected for all parameters, none of these plots indicated convergence problems. The difference (i.e., bias) between estimations with 1.5 million and 3.0 million iterations were minor.

Bayesian Plots

Figures S7 to S30 toward the end of these supplemental materials show traceplots, distribution plots and autocorrelation plots for all unstandardized Bayesian parameters (and R-squared coefficients). As shown in autocorrelation plots, some parameters had high autocorrelations from one iteration to the next. We solved this issue by using many iterations (1.5 million). We did not use thinning, which would dismiss valuable information (see Link and Eaton, 2012).

Table S15: Model 1, differences (bias) for point estimates with either 1.5 million or 3.0 million Bayesian iterations

	Older respondents			Younger respondents		
	1.5 mill	3.0 mill	Bias	1.5 mill	3.0 mill	Bias
**Individual level**						
Loading Prejudice	1.00	1.00	0.00	1.00	1.00	0
Loading Respect	1.91	1.91	0.00	2.09	2.10	0
Loading Treated	1.62	1.62	0.00	1.37	1.36	0
Female	0.11	0.10	0.00	0.07	0.07	0
Age	0.00	0.00	0.00	-0.04	-0.04	0
Residual Discrimination	2.45	2.45	0.00	1.77	1.77	0
**Country level**						
Loading Prejudice	1.00	1.00	0.00	1.00	1.00	0
Loading Respect	1.99	1.98	0.00	1.58	1.57	0
Loading Treated	1.96	1.96	0.00	0.64	0.64	0
AgeWatch Index	-0.12	-0.12	0.00	0.10	0.10	0
Th.hold 1 Prejudice	0.94	0.94	0.01	0.14	0.14	0
Th.hold 2 Prejudice	1.90	1.89	0.01	1.04	1.04	0
Th.hold 3 Prejudice	2.76	2.75	0.00	1.94	1.94	0
Th.hold 4 Prejudice	3.73	3.72	0.00	3.11	3.11	0
Th.hold 1 Respect	1.30	1.29	0.02	-0.04	-0.04	0
Th.hold 2 Respect	3.06	3.04	0.02	1.81	1.81	0
Th.hold 3 Respect	4.58	4.56	0.02	3.38	3.38	0
Th.hold 4 Respect	6.25	6.22	0.03	5.47	5.46	0
Th.hold 1 Treated	1.68	1.67	0.01	0.69	0.68	0
Th.hold 2 Treated	3.23	3.22	0.01	2.00	2.00	0
Th.hold 3 Treated	4.58	4.57	0.01	3.15	3.15	0
Th.hold 4 Treated	6.08	6.07	0.01	4.53	4.52	0
Residual Prejudice	0.05	0.05	0.00	0.05	0.05	0
Residual Respect	0.10	0.10	0.00	0.07	0.07	0
Residual Treated	0.02	0.02	0.00	0.10	0.10	0
Residual Discrimination	0.12	0.12	0.00	0.24	0.24	0

Note:
The table compares point estimates with 1.5 million and with 3.0 million iterations. Their difference is referred to as bias.

Doubling the Number of Bayesian Iterations

We doubled the number of Bayesian iterations, and also used a different seed number (`bseed = 543` instead of `bseed = 1234`, to test for local solutions). Tables S15 to S18 show bias, defined as the differences between point estimates obtained with 1.5 million and 3.0 million iterations. Bias was low, supporting the estimations using 1.5 million iterations.

Table S16: Model 2, differences (bias) for point estimates with either 1.5 million or 3.0 million Bayesian iterations

	Older respondents			Younger respondents		
	1.5 mill	3.0 mill	Bias	1.5 mill	3.0 mill	Bias
**Individual level**						
Loading Prejudice	1.00	1.00	0.00	1.00	1.00	0
Loading Respect	1.91	1.91	0.00	2.09	2.10	0
Loading Treated	1.62	1.62	0.00	1.37	1.36	0
Female	0.10	0.10	0.00	0.07	0.07	0
Age	0.00	0.00	0.00	-0.04	-0.04	0
Residual Discrimination	2.45	2.45	0.00	1.77	1.77	0
**Country level**						
Loading Prejudice	1.00	1.00	0.00	1.00	1.00	0
Loading Respect	1.97	1.97	0.00	1.62	1.62	0
Loading Treated	1.91	1.91	0.00	0.68	0.68	0
AgeWatch Index	-0.53	-0.53	0.00	0.07	0.07	0
Th.hold 1 Prejudice	0.92	0.91	0.01	0.11	0.11	0
Th.hold 2 Prejudice	1.87	1.86	0.01	1.01	1.01	0
Th.hold 3 Prejudice	2.74	2.73	0.01	1.91	1.91	0
Th.hold 4 Prejudice	3.70	3.69	0.01	3.09	3.09	0
Th.hold 1 Respect	1.26	1.24	0.03	-0.08	-0.09	0
Th.hold 2 Respect	3.02	2.99	0.03	1.77	1.76	0
Th.hold 3 Respect	4.54	4.51	0.03	3.34	3.34	0
Th.hold 4 Respect	6.21	6.17	0.03	5.42	5.42	0
Th.hold 1 Treated	1.64	1.62	0.02	0.67	0.67	0
Th.hold 2 Treated	3.19	3.16	0.02	1.98	1.98	0
Th.hold 3 Treated	4.54	4.52	0.02	3.13	3.13	0
Th.hold 4 Treated	6.04	6.02	0.02	4.51	4.50	0
Residual Prejudice	0.04	0.04	0.00	0.06	0.06	0
Residual Respect	0.09	0.09	0.00	0.05	0.05	0
Residual Treated	0.04	0.04	0.00	0.09	0.09	0
Residual Discrimination	0.16	0.17	0.00	0.37	0.37	0

Note:
The table compares point estimates with 1.5 million and with 3.0 million iterations. Their difference is referred to as bias.

Table S17: Model 3, differences (bias) for point estimates with either 1.5 million or 3.0 million Bayesian iterations

	Older respondents			Younger respondents		
	1.5 mill	3.0 mill	Bias	1.5 mill	3.0 mill	Bias
**Individual level**						
Loading Prejudice	1.00	1.00	0	1.00	1.00	0.00
Loading Respect	1.91	1.91	0	2.09	2.09	0.00
Loading Treated	1.62	1.62	0	1.36	1.36	0.00
Female	0.10	0.10	0	0.07	0.07	0.00
Age	0.00	0.00	0	-0.04	-0.04	0.00
Residual Discrimination	2.45	2.45	0	1.77	1.77	0.00
**Country level**						
Loading Prejudice	1.00	1.00	0	1.00	1.00	0.00
Loading Respect	1.96	1.96	0	1.55	1.55	0.00
Loading Treated	1.93	1.93	0	0.63	0.63	0.00
AgeWatch Index	-0.08	-0.08	0	0.13	0.13	0.00
Social norm	-0.29	-0.28	0	-0.29	-0.29	0.00
Th.hold 1 Prejudice	0.94	0.94	0	0.13	0.14	-0.01
Th.hold 2 Prejudice	1.90	1.90	0	1.03	1.04	0.00
Th.hold 3 Prejudice	2.76	2.76	0	1.93	1.94	-0.01
Th.hold 4 Prejudice	3.73	3.73	0	3.11	3.11	-0.01
Th.hold 1 Respect	1.31	1.30	0	-0.05	-0.04	-0.01
Th.hold 2 Respect	3.06	3.05	0	1.80	1.81	-0.01
Th.hold 3 Respect	4.58	4.58	0	3.37	3.38	-0.01
Th.hold 4 Respect	6.24	6.25	0	5.46	5.46	-0.01
Th.hold 1 Treated	1.68	1.68	0	0.68	0.68	0.00
Th.hold 2 Treated	3.23	3.23	0	1.99	2.00	0.00
Th.hold 3 Treated	4.58	4.58	0	3.14	3.15	0.00
Th.hold 4 Treated	6.08	6.08	0	4.52	4.52	0.00
Residual Prejudice	0.05	0.05	0	0.04	0.04	0.00
Residual Respect	0.10	0.10	0	0.08	0.08	0.00
Residual Treated	0.02	0.02	0	0.10	0.10	0.00
Residual Discrimination	0.10	0.10	0	0.22	0.22	0.00

Note:
The table compares point estimates with 1.5 million and with 3.0 million iterations. Their difference is referred to as bias.

Table S18: Model 4, differences (bias) for point estimates with either 1.5 million or 3.0 million Bayesian iterations

	Older respondents			Younger respondents		
	1.5 mill	3.0 mill	Bias	1.5 mill	3.0 mill	Bias
**Individual level**						
Loading Prejudice	1.00	1.00	0.00	1.00	1.00	0.00
Loading Respect	1.91	1.90	0.01	2.10	2.09	0.00
Loading Treated	1.62	1.62	0.00	1.36	1.36	0.00
Female	0.10	0.10	0.00	0.07	0.07	0.00
Age	0.00	0.00	0.00	-0.04	-0.04	0.00
Residual Discrimination	2.45	2.46	0.00	1.77	1.77	0.00
**Country level**						
Loading Prejudice	1.00	1.00	0.00	1.00	1.00	0.00
Loading Respect	1.94	1.94	0.01	1.54	1.54	0.00
Loading Treated	1.92	1.92	0.00	0.63	0.62	0.00
AgeWatch Index	-0.08	-0.08	0.00	0.12	0.12	0.00
Social norm	-0.36	-0.36	0.00	-0.18	-0.17	0.00
Interaction	-0.03	-0.03	0.00	0.06	0.06	0.00
Th.hold 1 Prejudice	0.90	0.91	0.00	0.21	0.22	-0.01
Th.hold 2 Prejudice	1.85	1.86	0.00	1.11	1.12	-0.01
Th.hold 3 Prejudice	2.72	2.72	0.00	2.01	2.02	-0.01
Th.hold 4 Prejudice	3.69	3.69	0.00	3.19	3.19	-0.01
Th.hold 1 Respect	1.22	1.22	0.00	0.07	0.08	-0.01
Th.hold 2 Respect	2.97	2.97	0.00	1.92	1.93	-0.01
Th.hold 3 Respect	4.49	4.49	0.00	3.49	3.50	0.00
Th.hold 4 Respect	6.16	6.16	0.00	5.58	5.58	0.00
Th.hold 1 Treated	1.60	1.60	0.00	0.73	0.73	0.00
Th.hold 2 Treated	3.15	3.15	-0.01	2.04	2.04	0.00
Th.hold 3 Treated	4.50	4.51	-0.01	3.19	3.19	-0.01
Th.hold 4 Treated	6.00	6.00	-0.01	4.56	4.57	-0.01
Residual Prejudice	0.05	0.05	0.00	0.04	0.04	0.00
Residual Respect	0.10	0.10	0.00	0.08	0.08	0.00
Residual Treated	0.02	0.02	0.00	0.10	0.10	0.00
Residual Discrimination	0.11	0.11	0.00	0.20	0.20	0.00

Note:
The table compares point estimates with 1.5 million and with 3.0 million iterations. Their difference is referred to as bias.

Plots of Bayesian Parameters

Below are trace plots, distribution plots, and autocorrelation plots for all Bayesian estimations (Models 1 to 4, for both older and younger respondents). The figures include plots of unstandardised estimates and R^2. The order of figures is as follows:

- Older repondents, Model 1 (pages 32 to 34)
- Older repondents, Model 2 (pages 35 to 37)
- Older repondents, Model 3 (pages 38 to 40)
- Older repondents, Model 4 (pages 41 to 43)
- Younger repondents, Model 1 (pages 44 to 46)
- Younger repondents, Model 2 (pages 47 to 49)
- Younger repondents, Model 3 (pages 50 to 52)
- Younger repondents, Model 4 (pages 53 to 55)

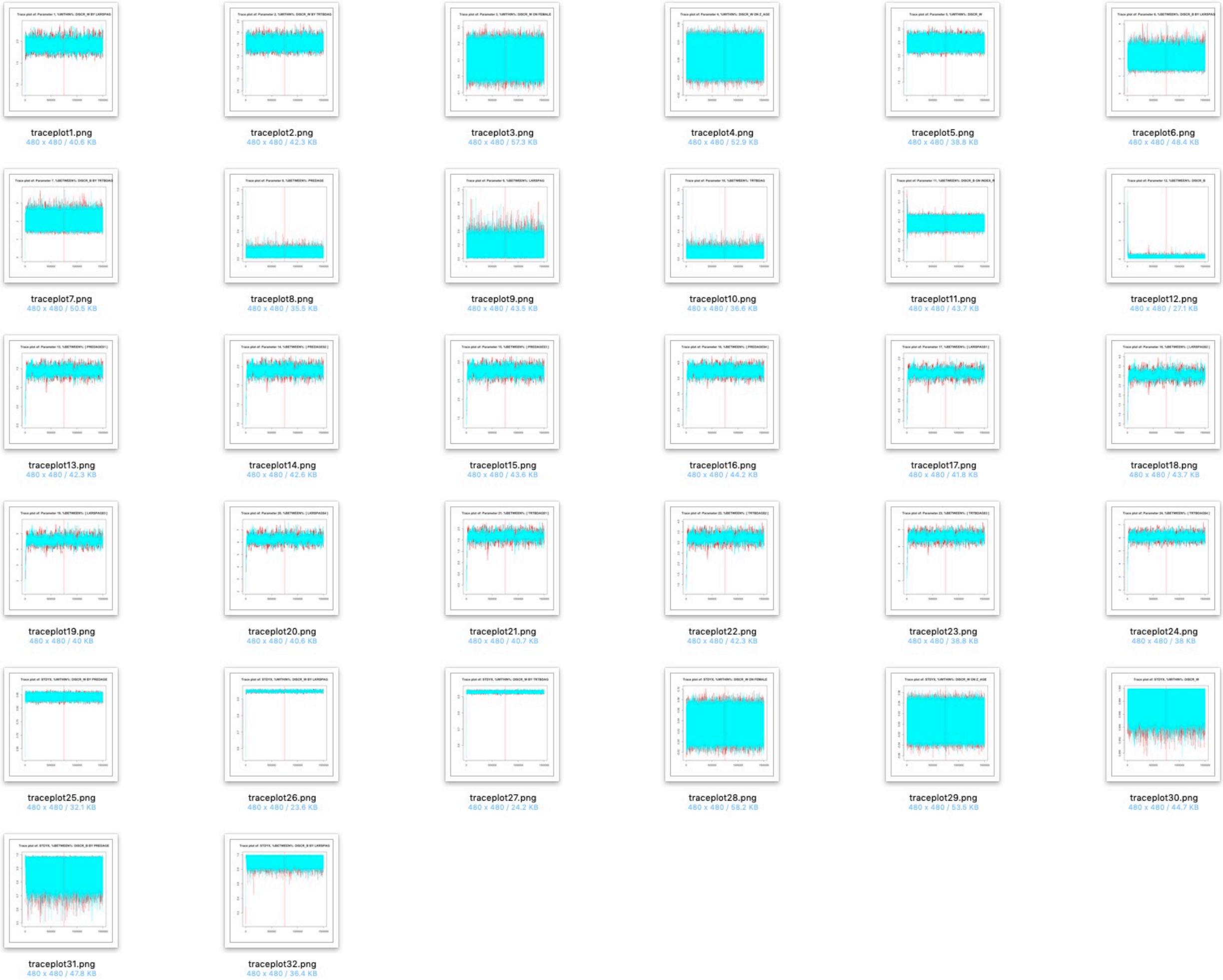

Figure S7: Older respondents, Model 1, trace plots

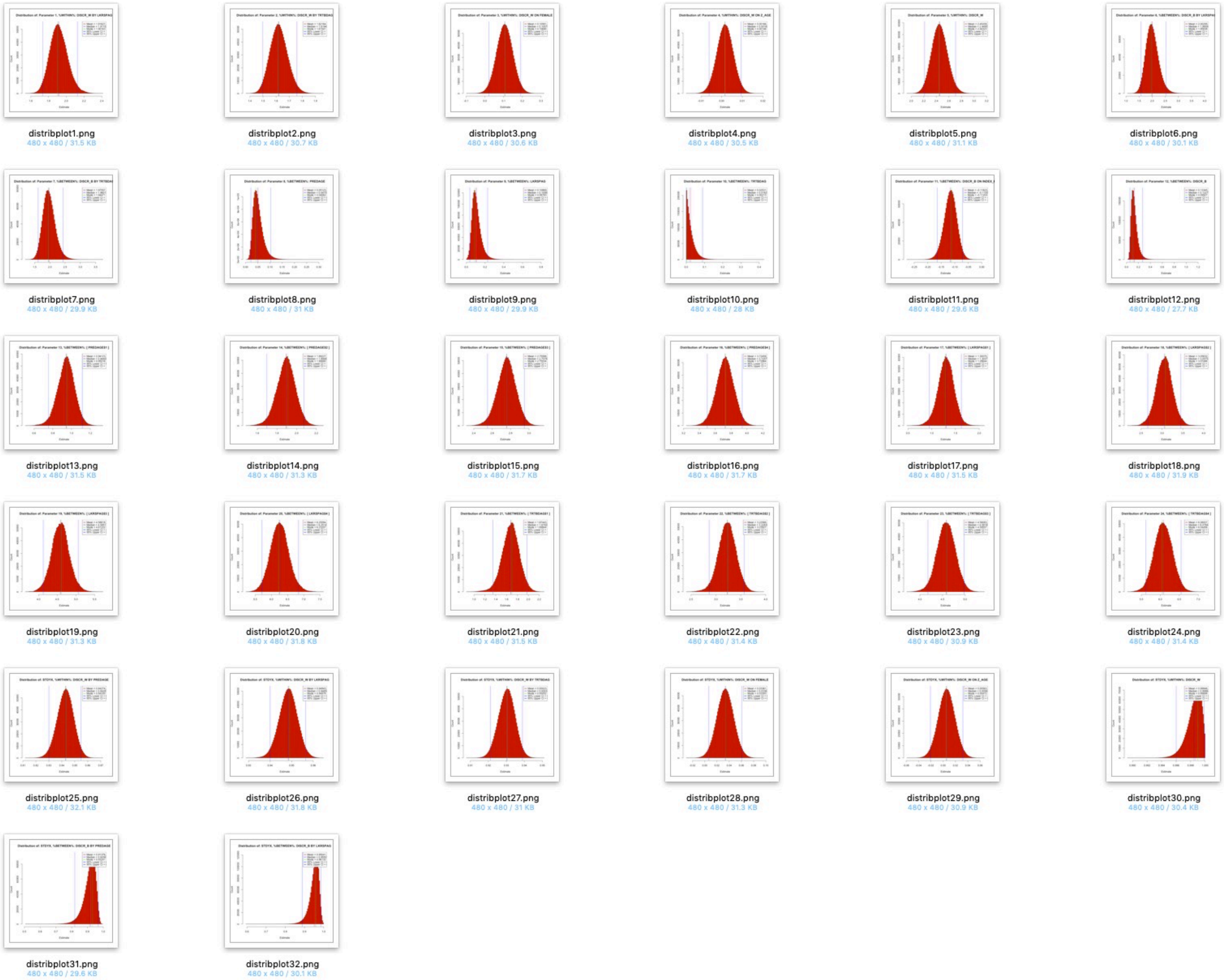

Figure S8: Older respondents, Model 1, distribution plots

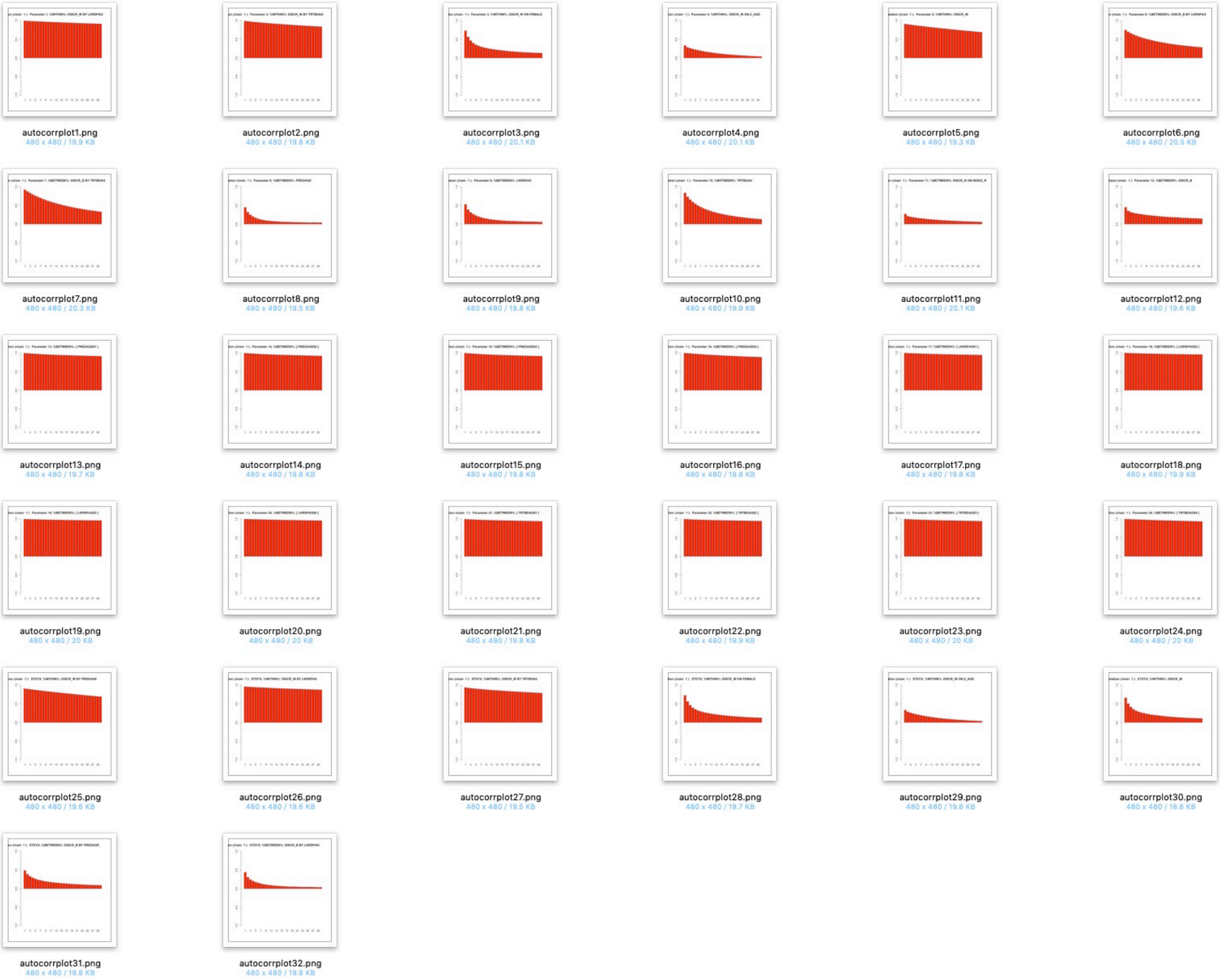

Figure S9: Older respondents, Model 1, autocorrelation plots

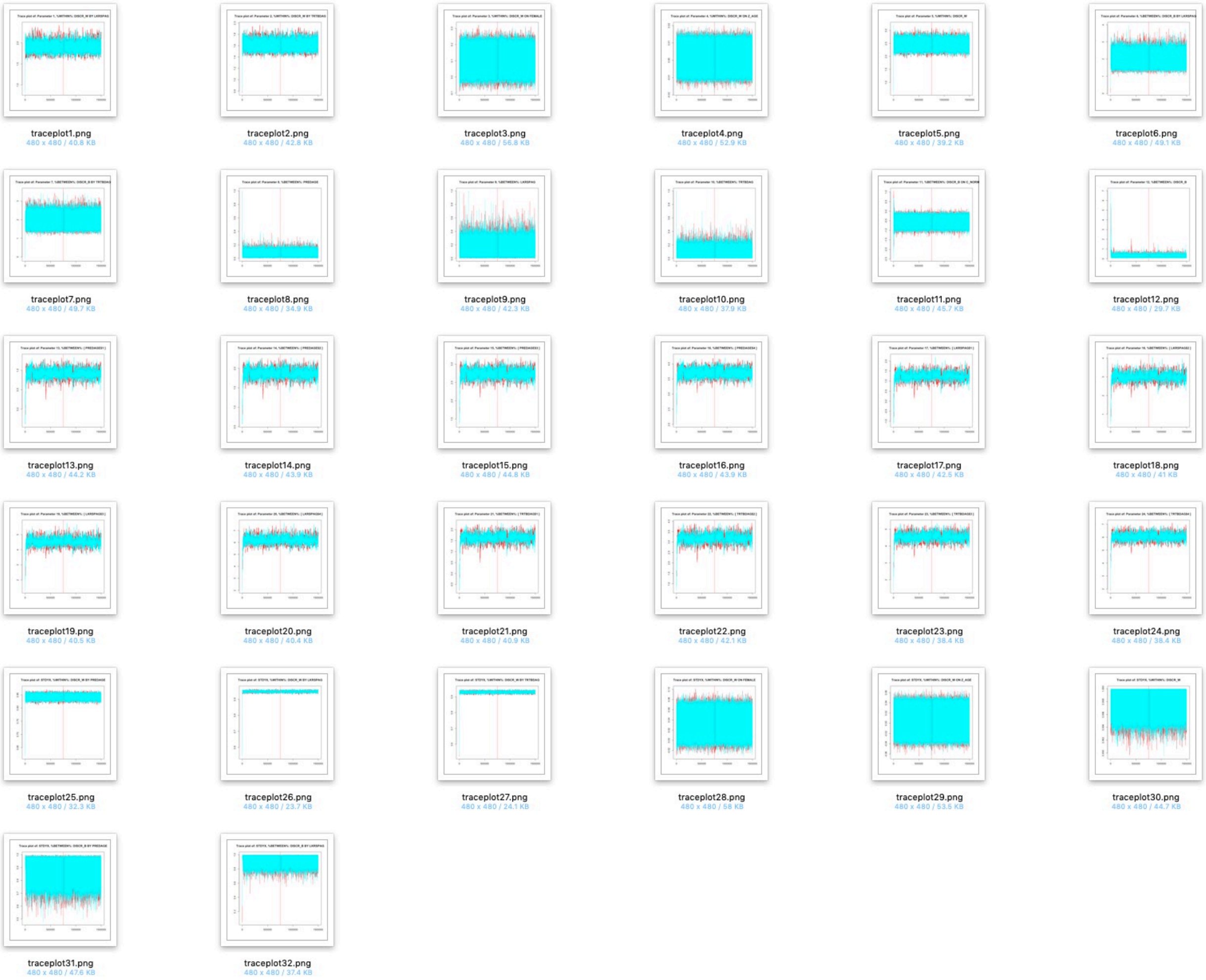

Figure S10: Older respondents, Model 2, trace plots

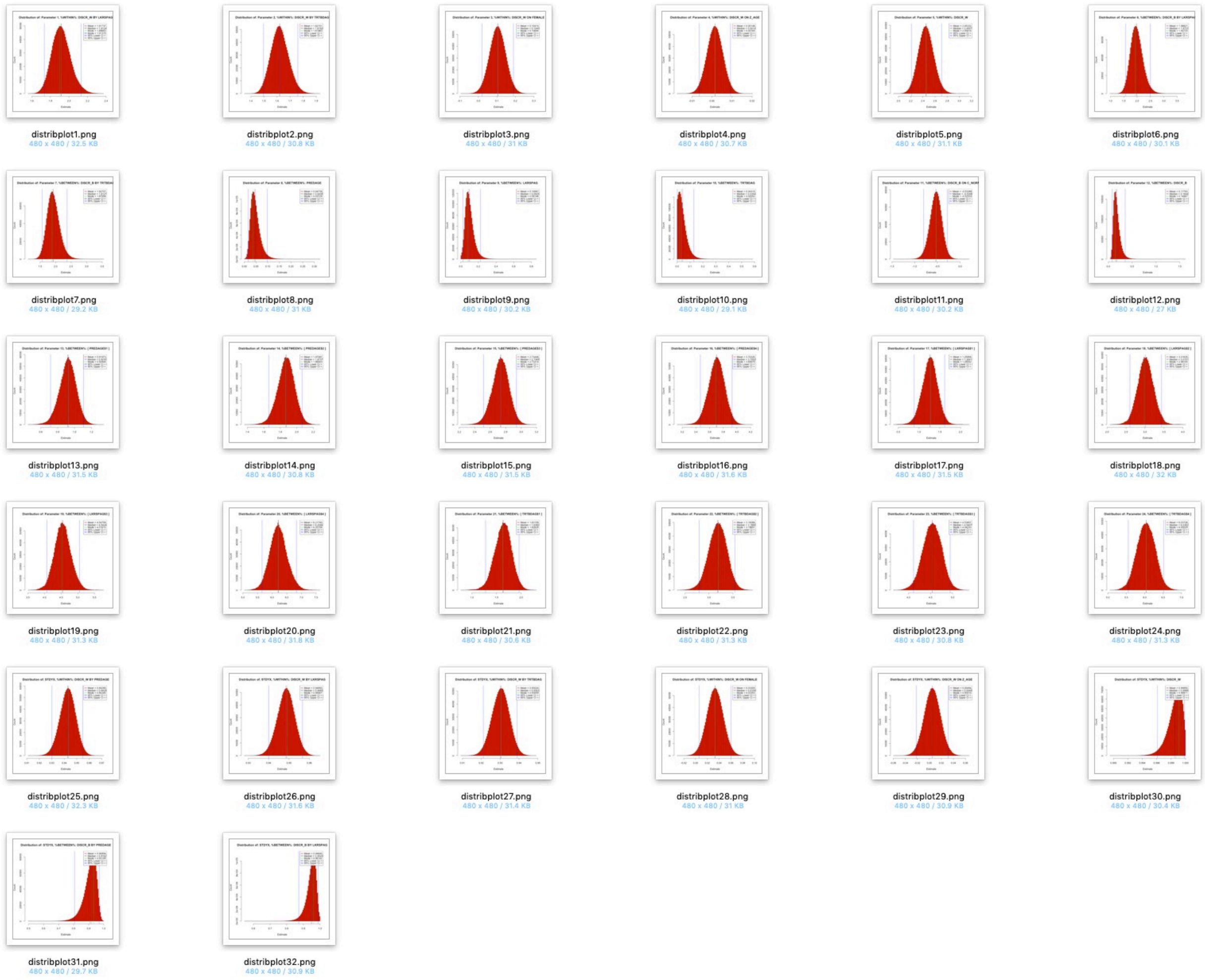

Figure S11: Older respondents, Model 2, distribution plots

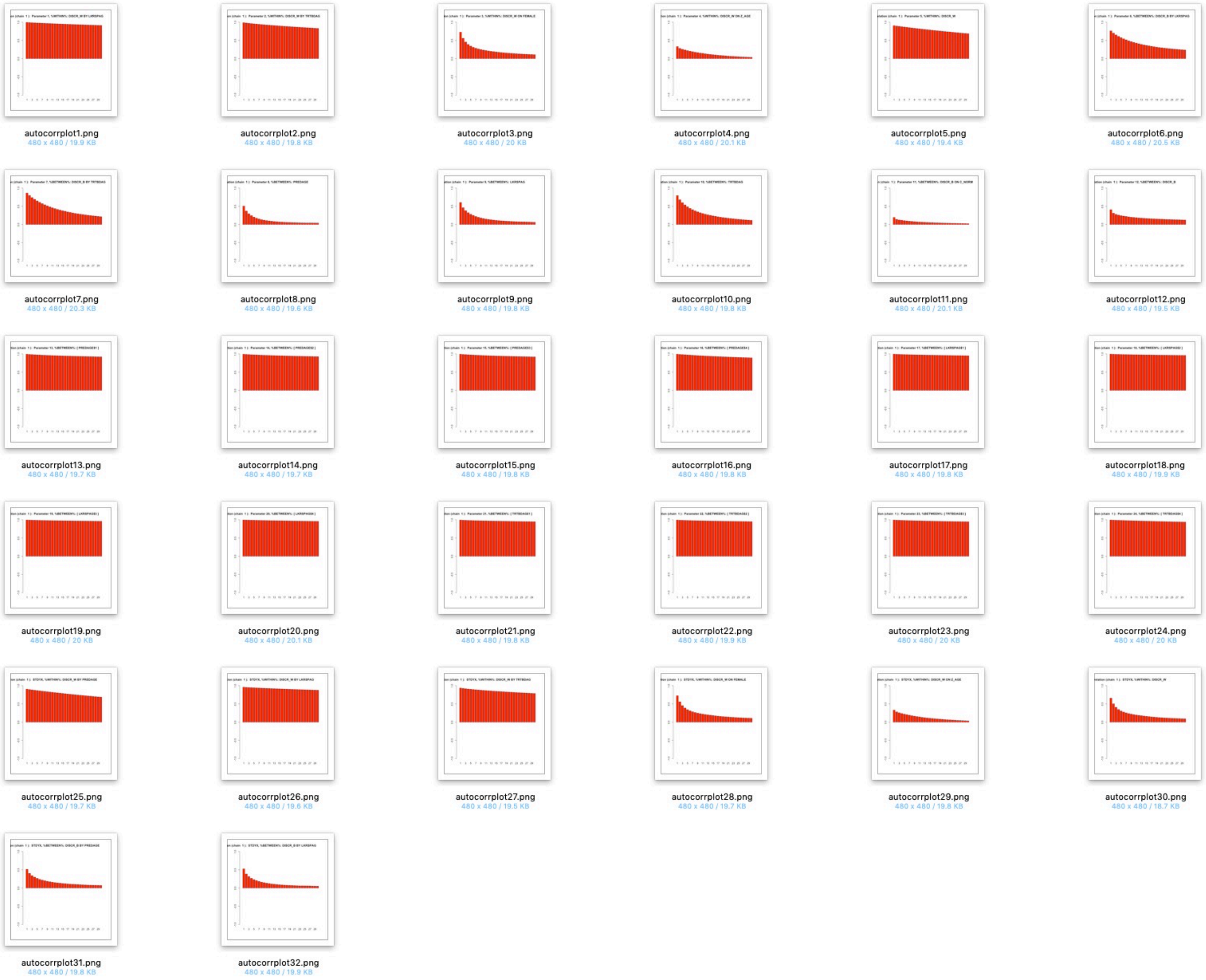

Figure S12: Older respondents, Model 2, autocorrelation plots

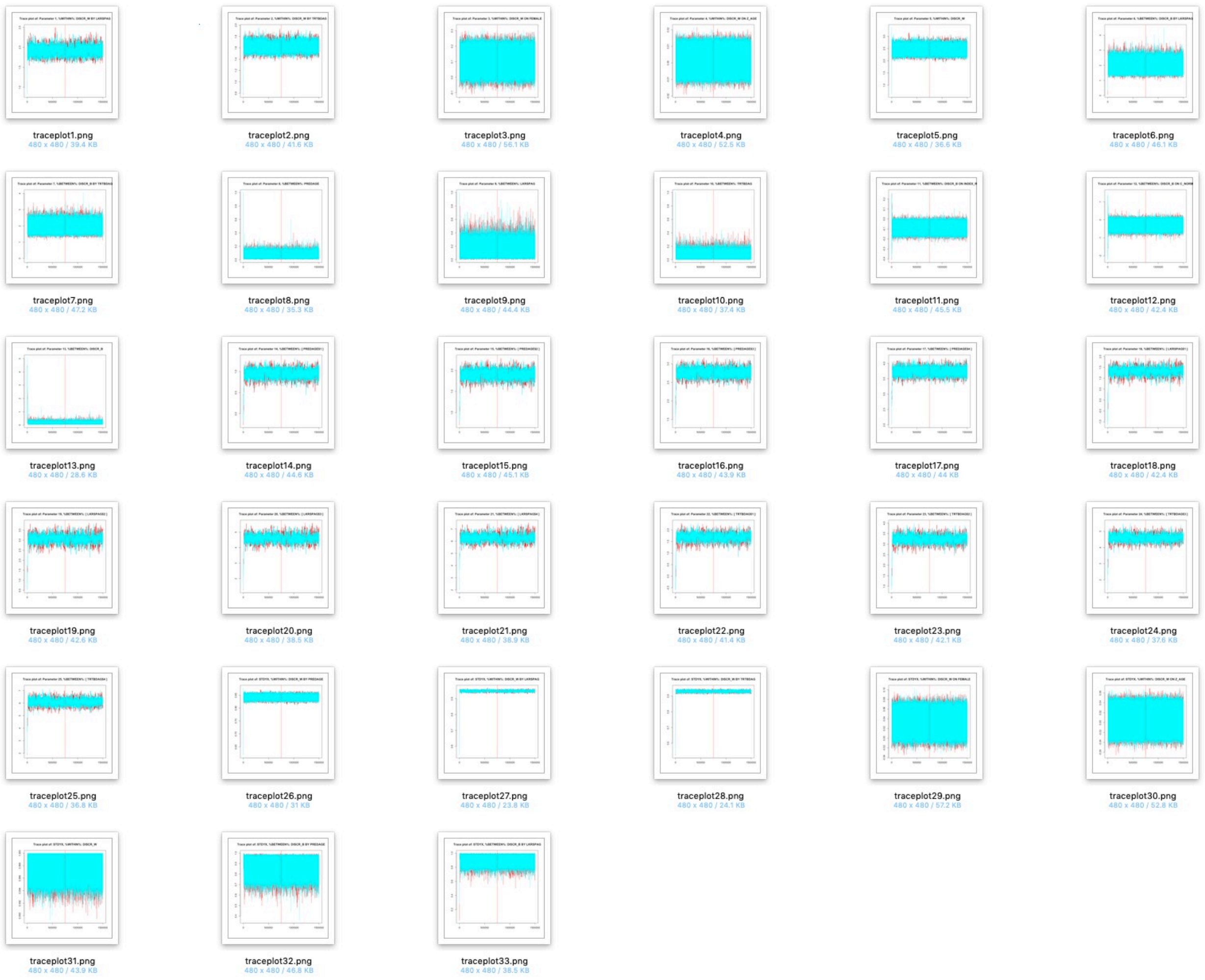

Figure S13: Older respondents, Model 3, trace plots

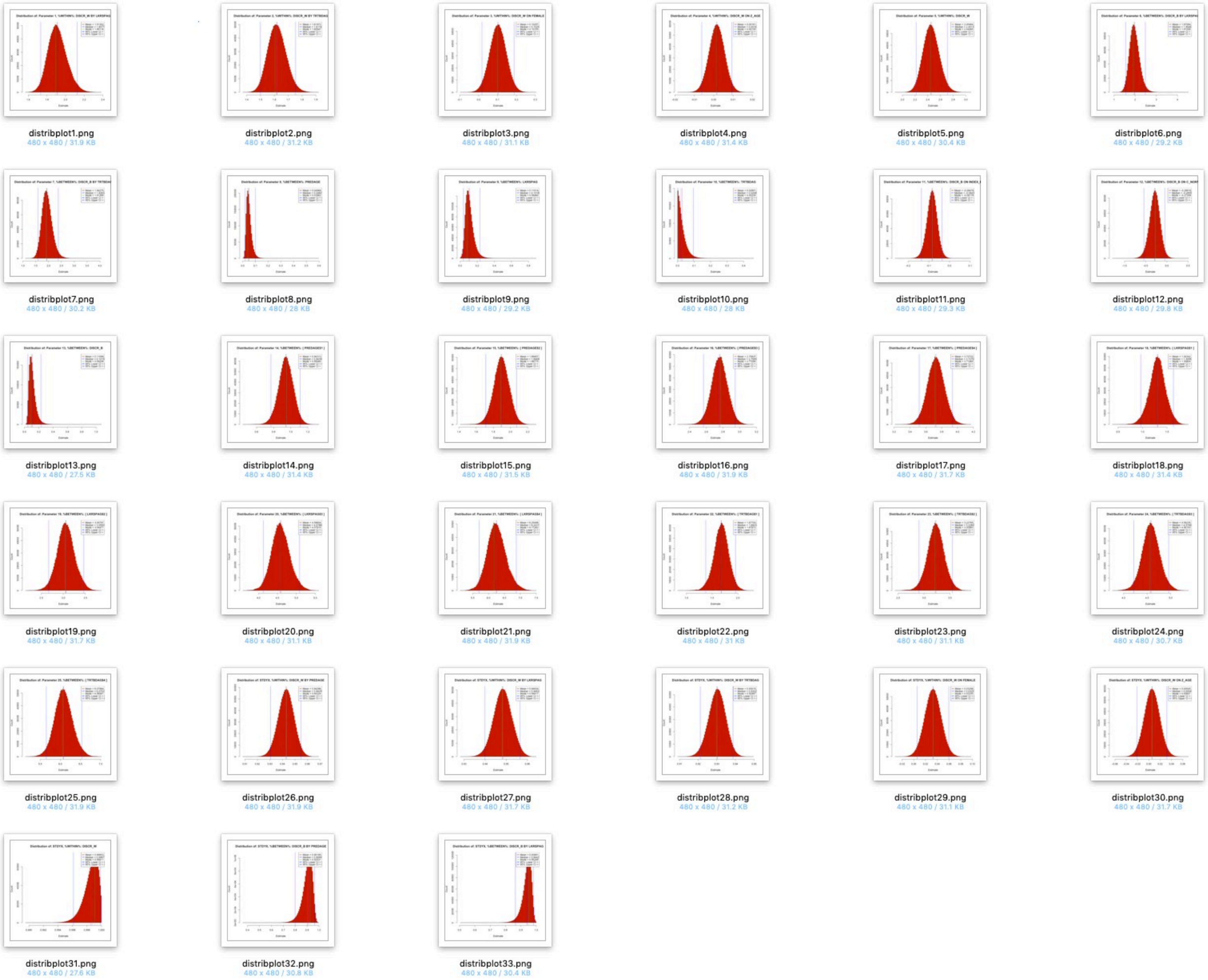

Figure S14: Older respondents, Model 3, distribution plots

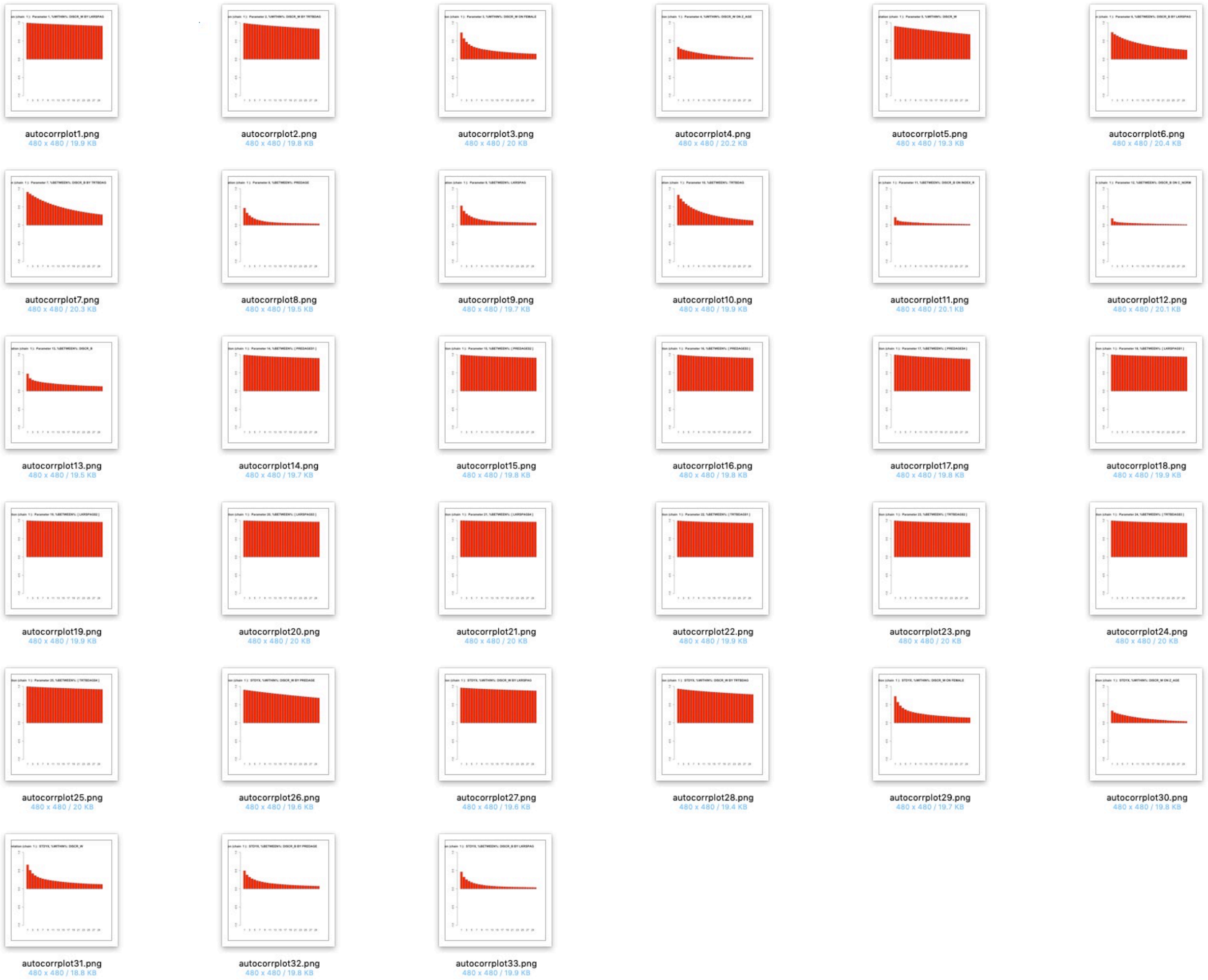

Figure S15: Older respondents, Model 3, autocorrelation plots

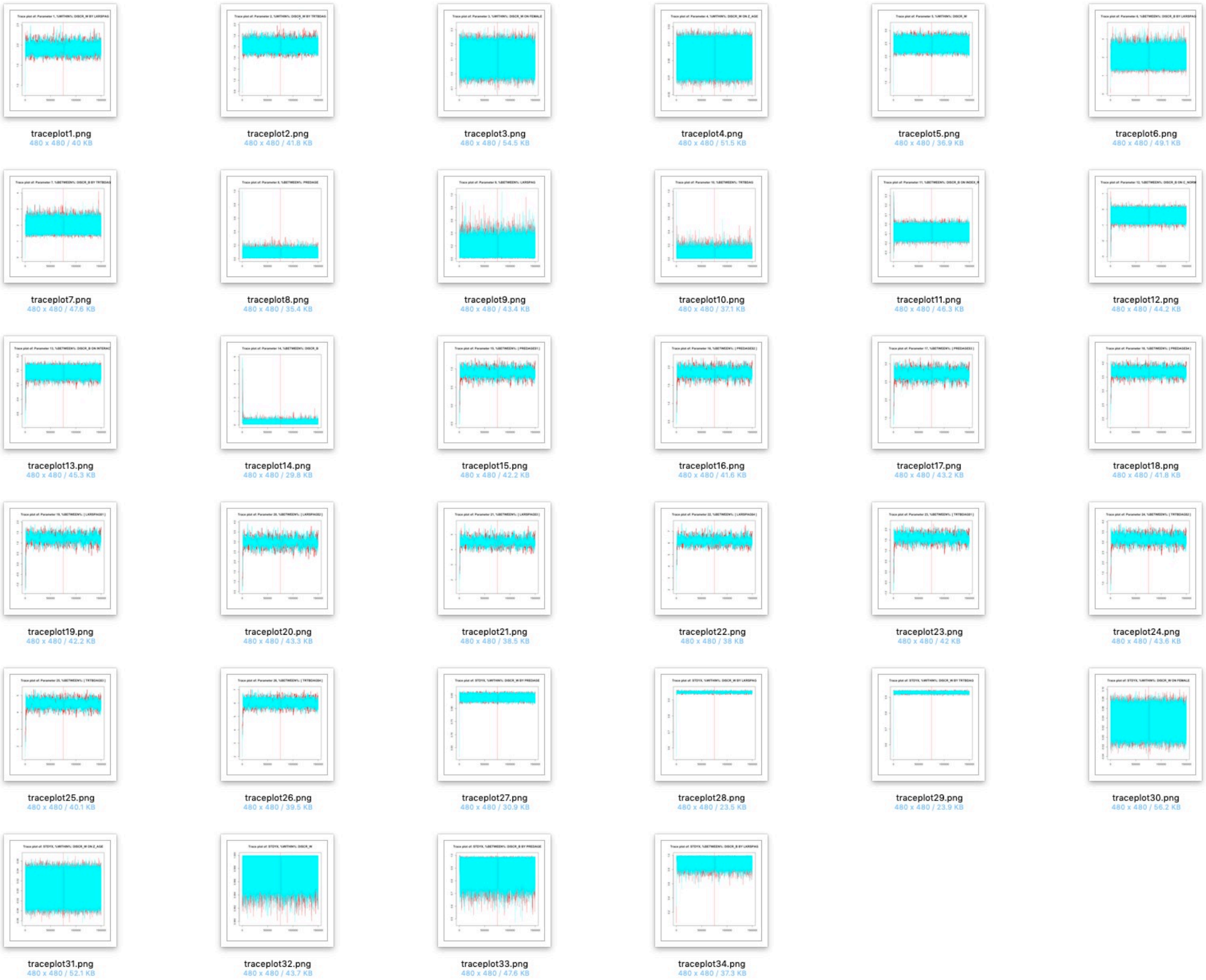

Figure S16: Older respondents, Model 4, trace plots

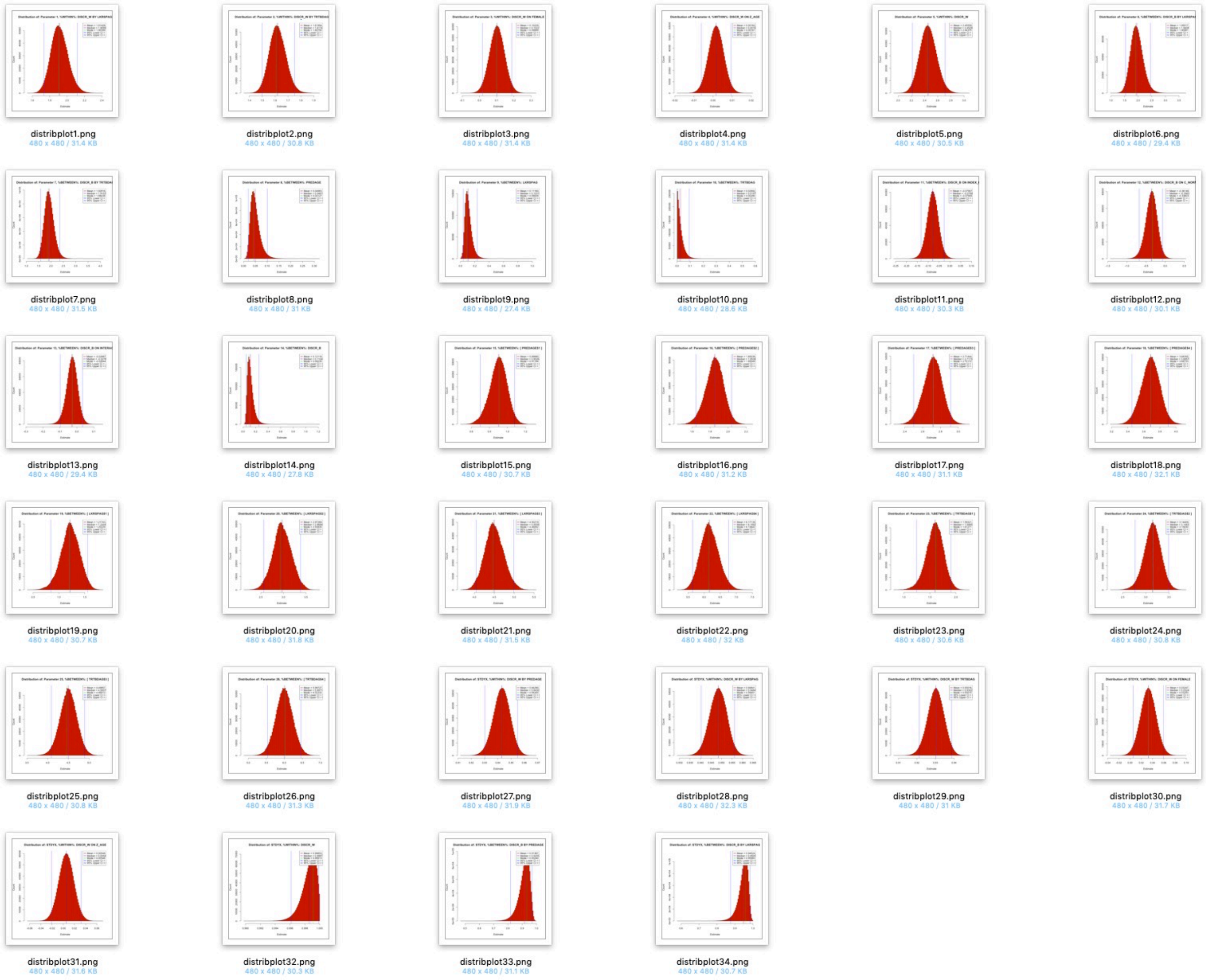

Figure S17: Older respondents, Model 4, distribution plots

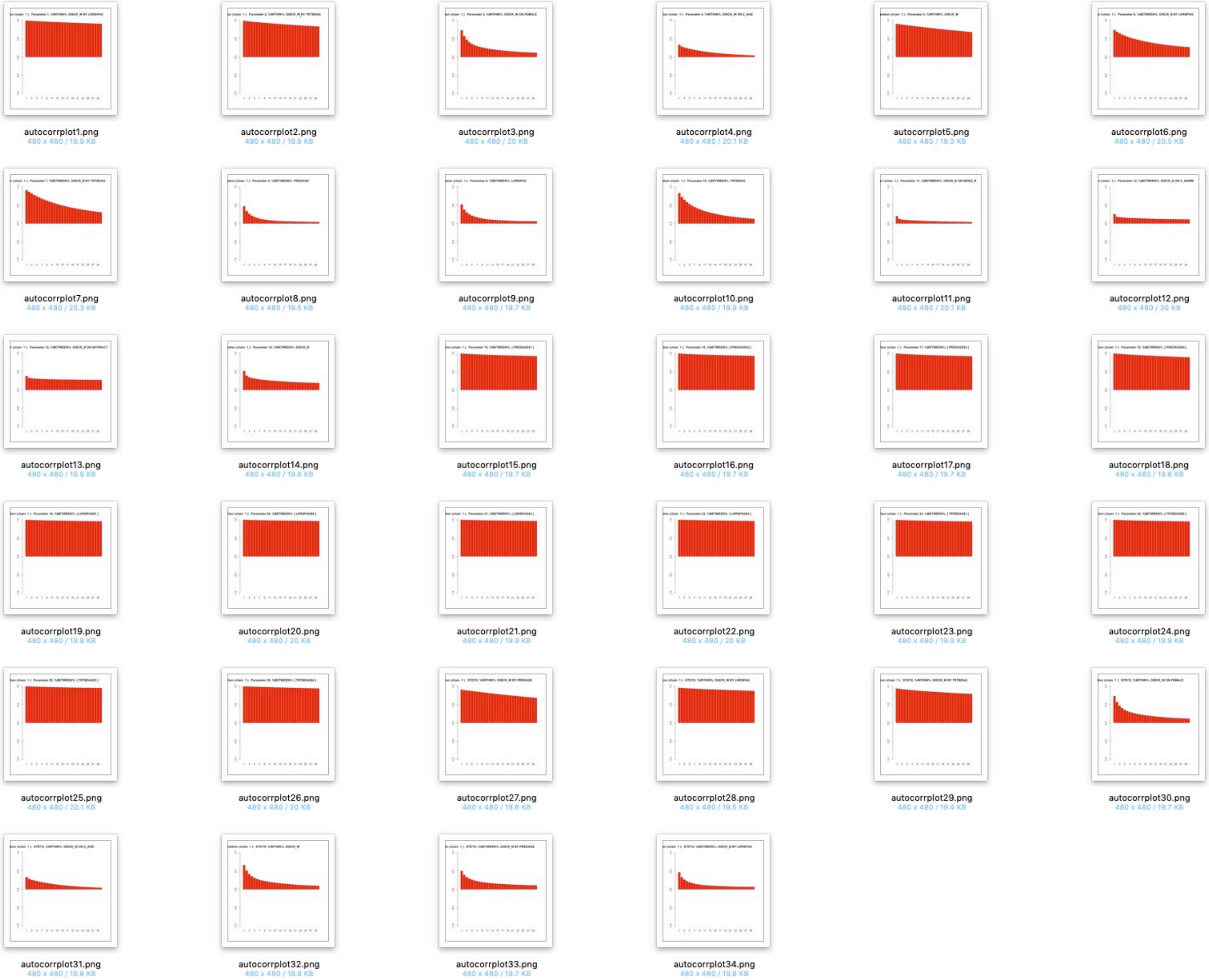

Figure S18: Older respondents, Model 4, autocorrelation plots

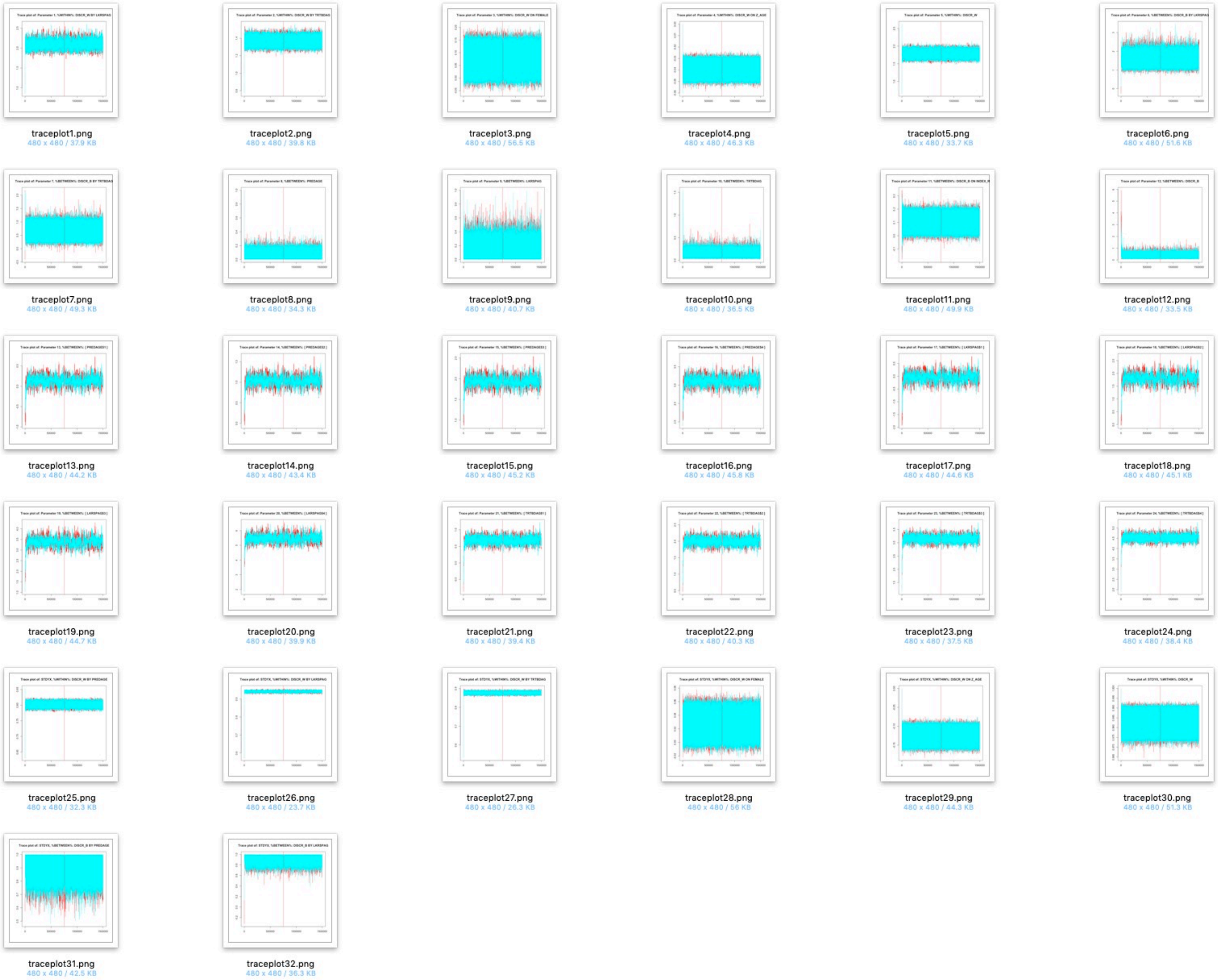

Figure S19: Younger respondents, Model 1, trace plots

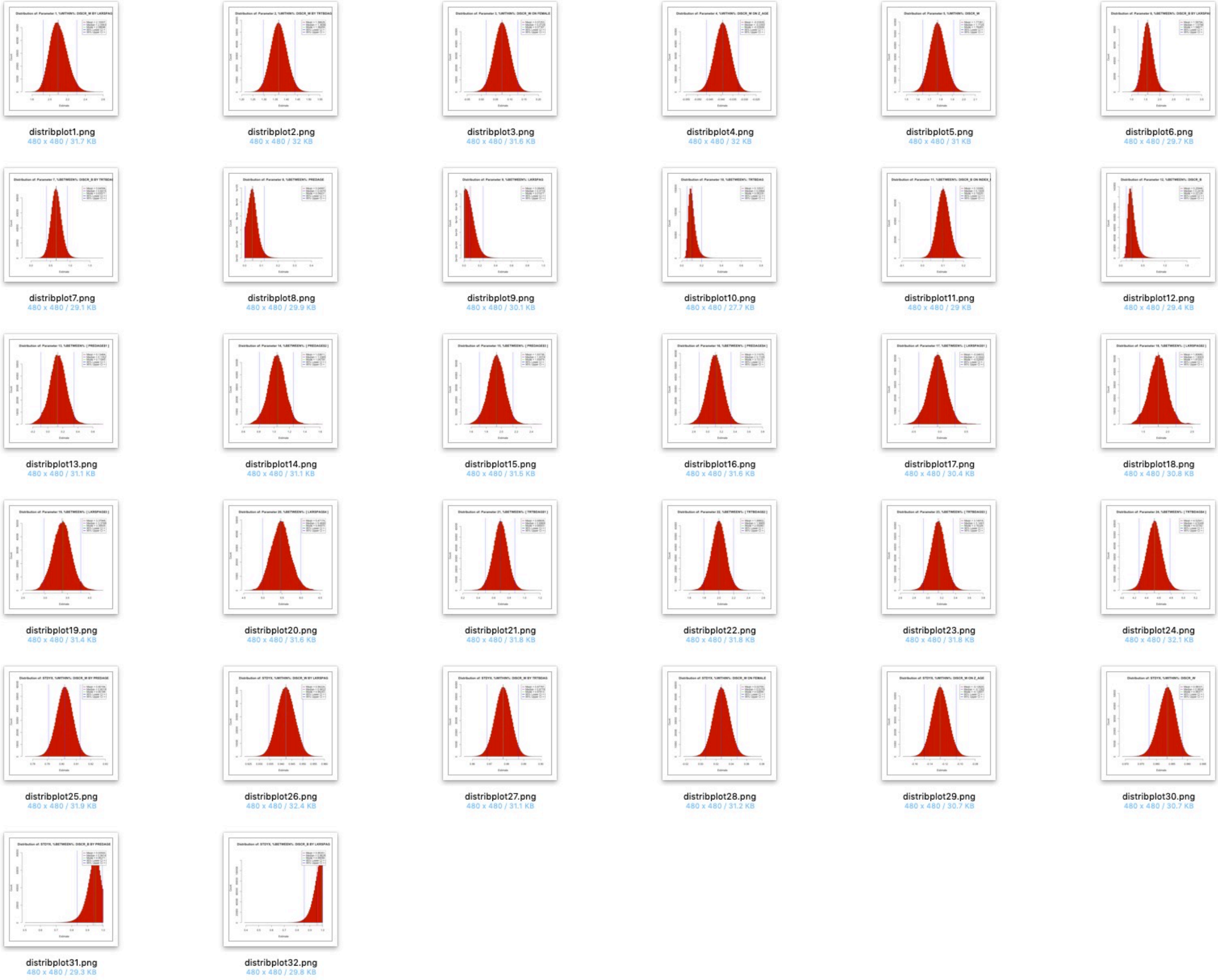

Figure S20: Younger respondents, Model 1, distribution plots

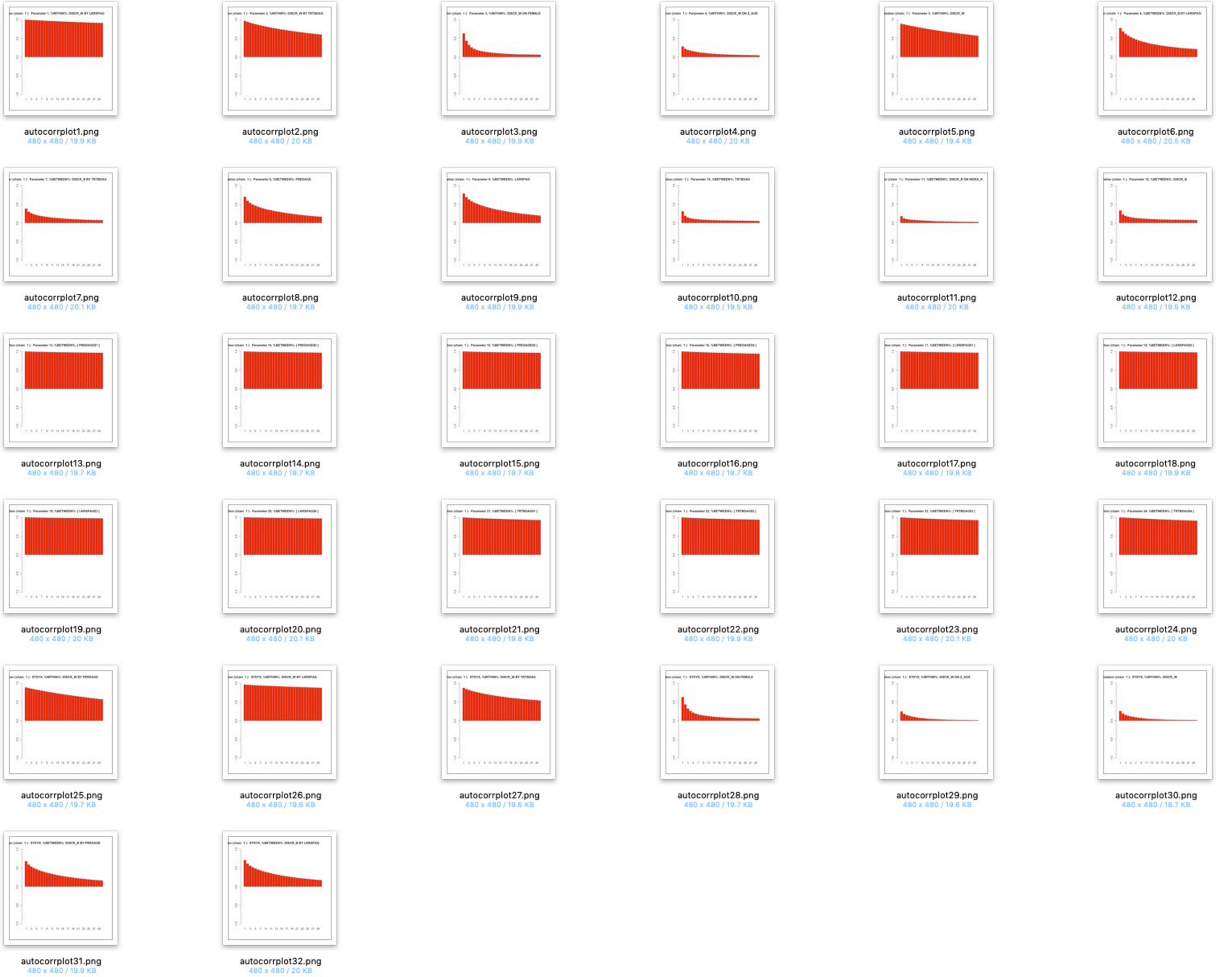

Figure S21: Younger respondents, Model 1, autocorrelation plots

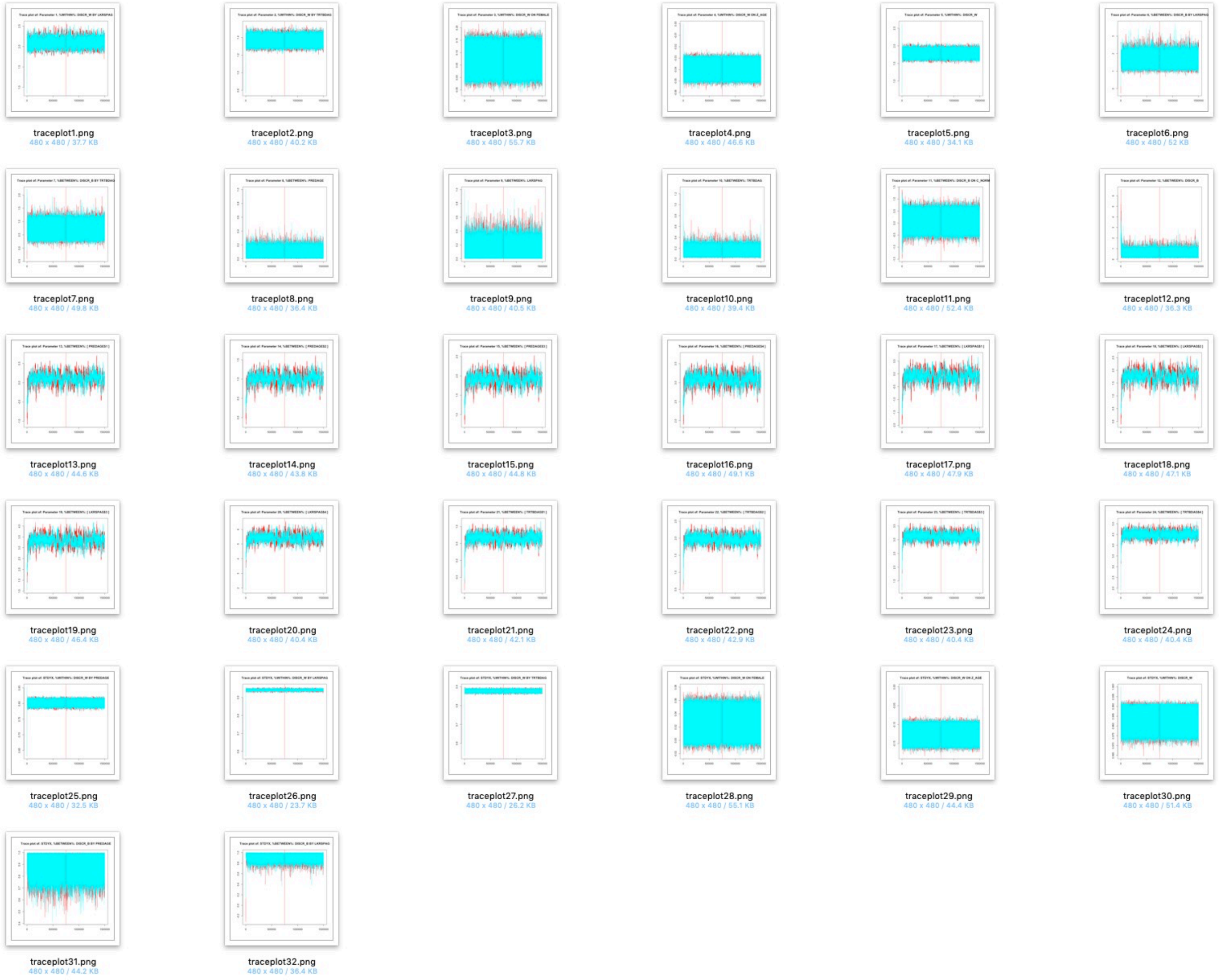

Figure S22: Younger respondents, Model 2, trace plots

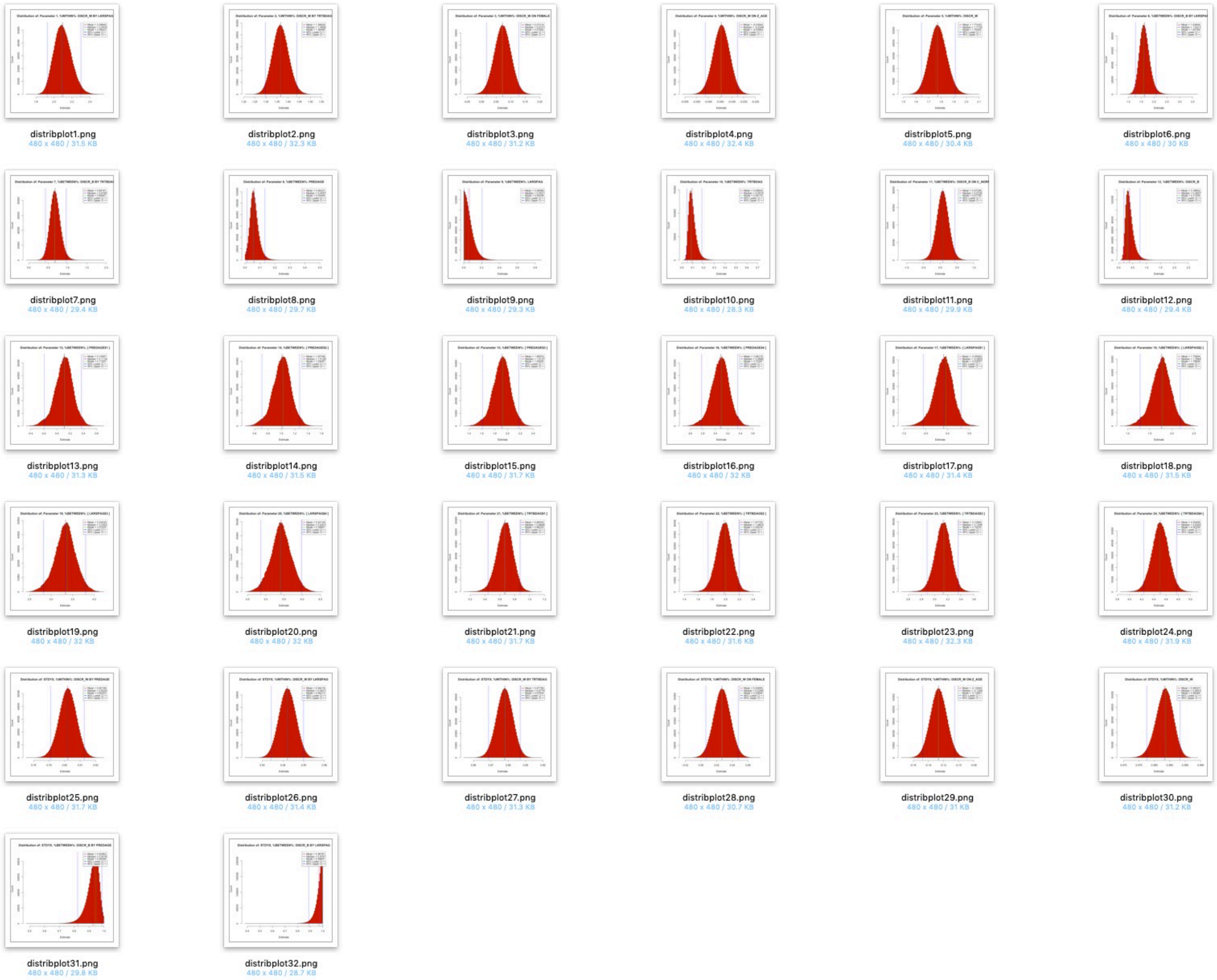

Figure S23: Younger respondents, Model 2, distribution plots

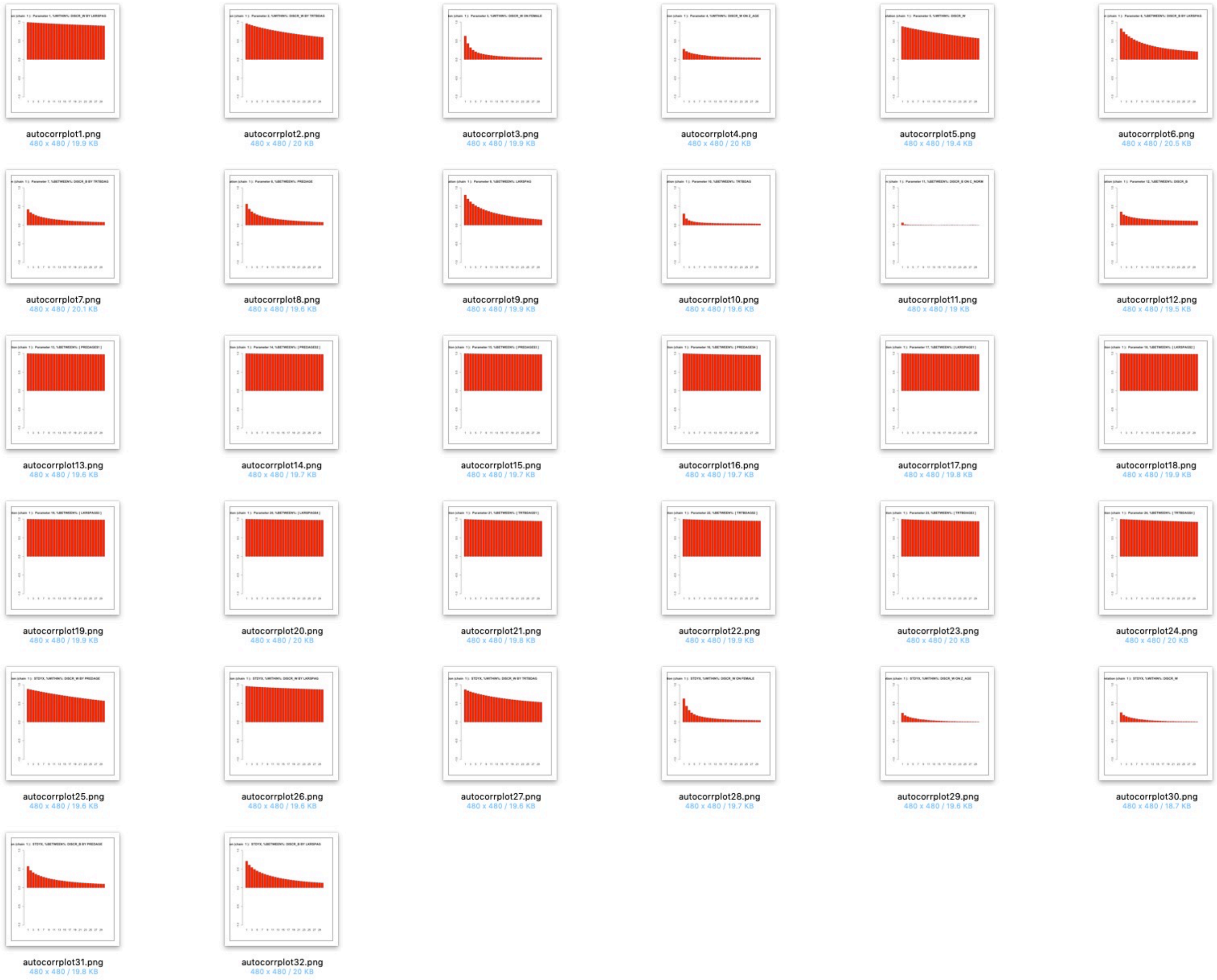

Figure S24: Younger respondents, Model 2, autocorrelation plots

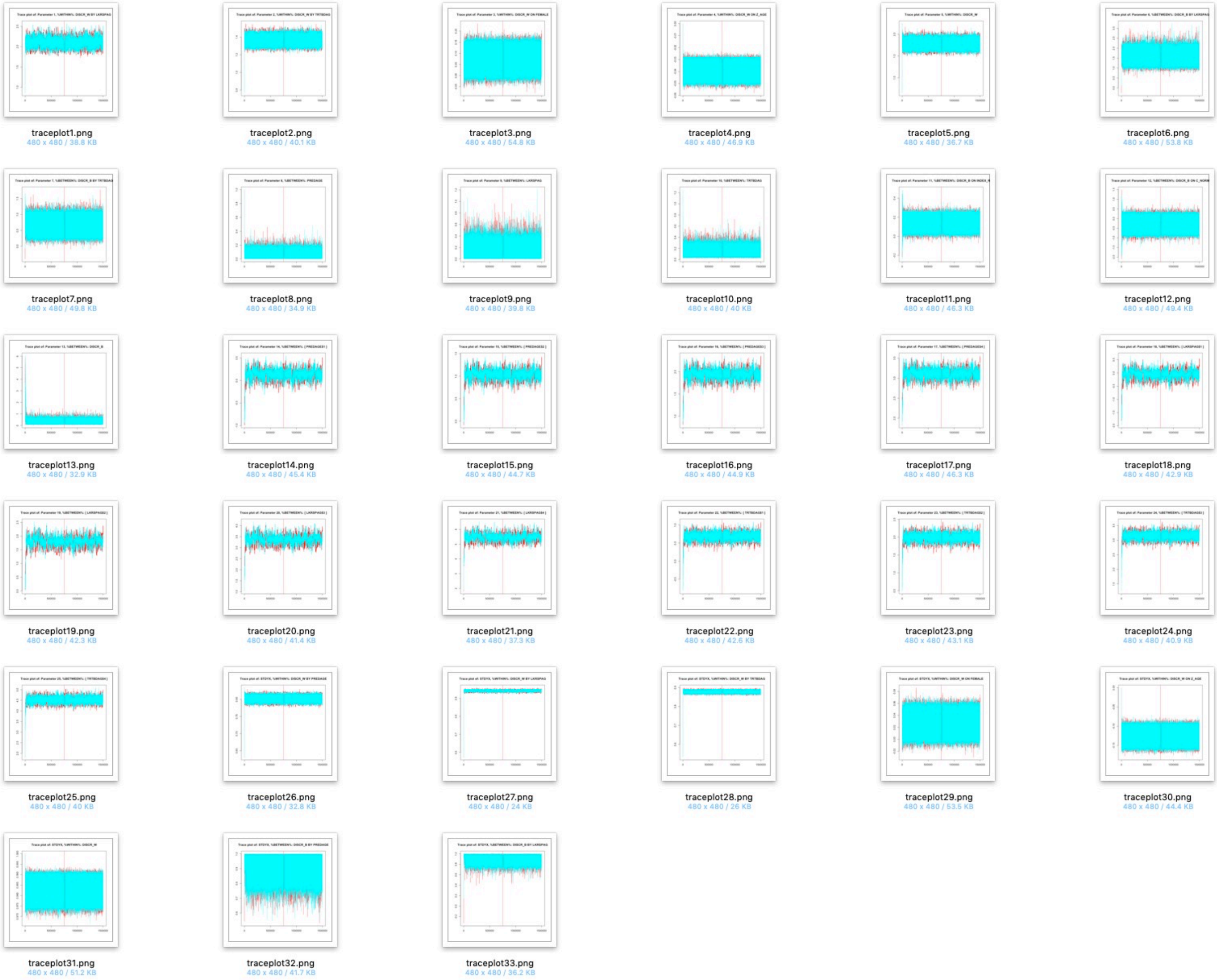

Figure S25: Younger respondents, Model 3, trace plots

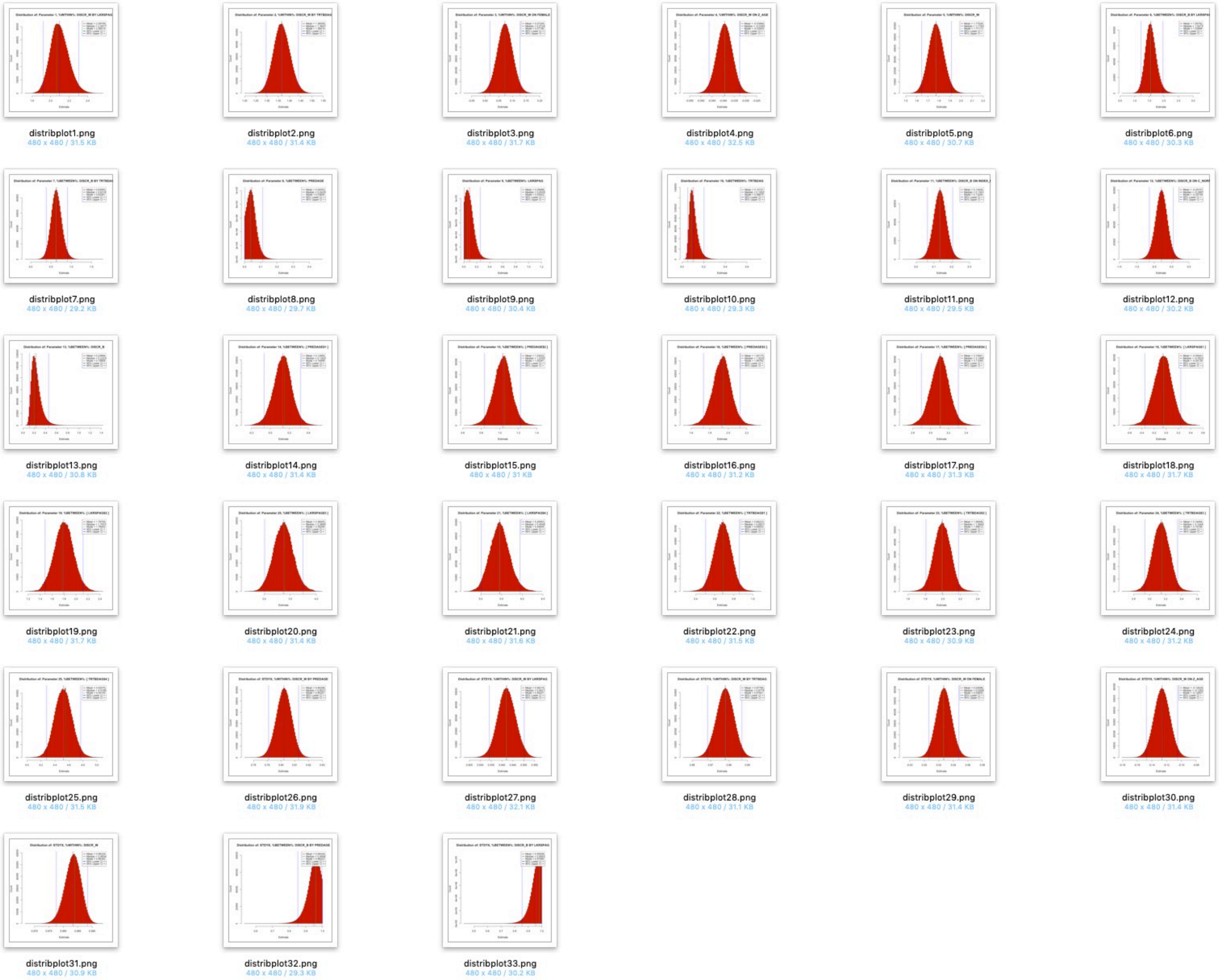

Figure S26: Younger respondents, Model 3, distribution plots

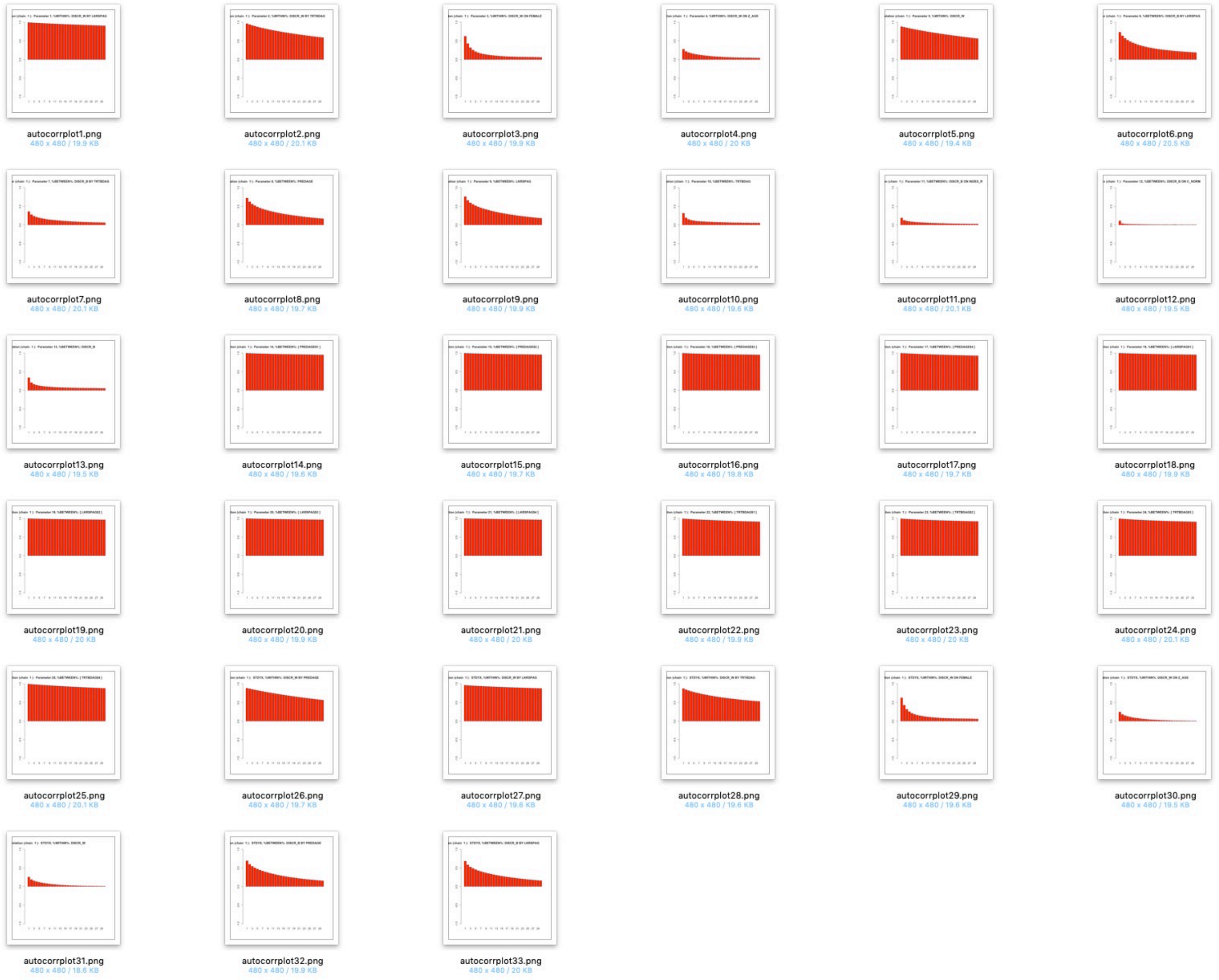

Figure S27: Younger respondents, Model 3, autocorrelation plots

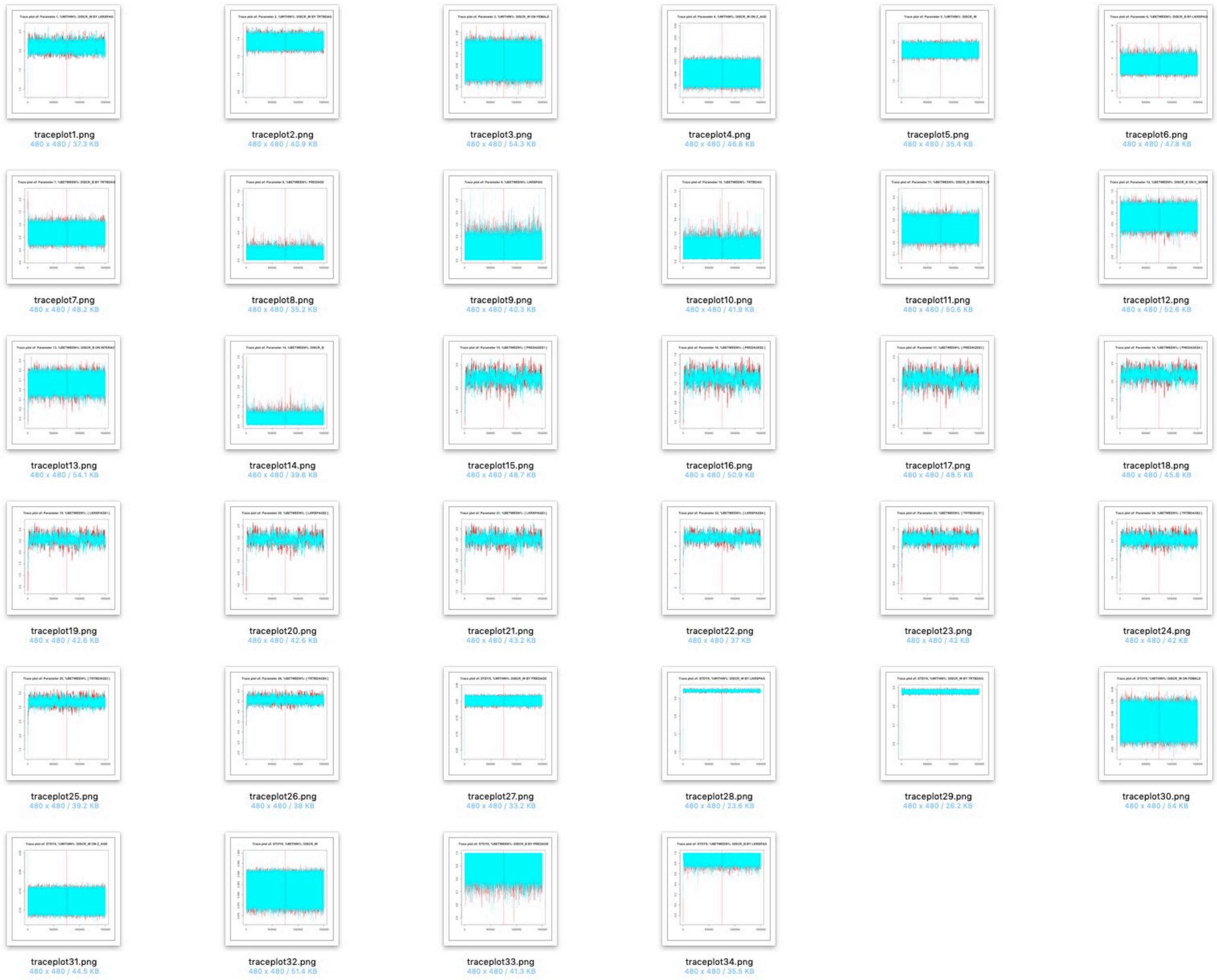

Figure S28: Younger respondents, Model 4, trace plots

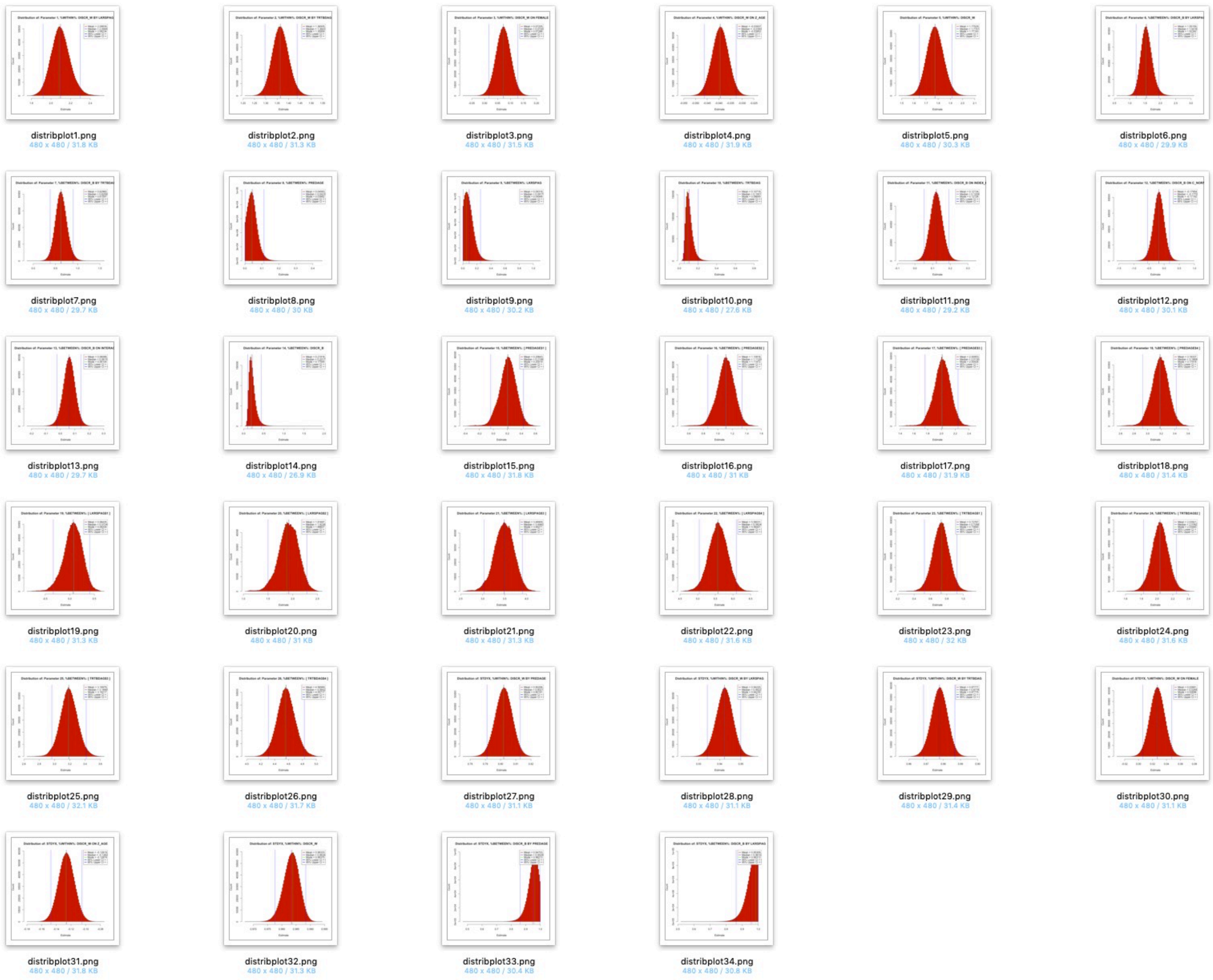

Figure S29: Younger respondents, Model 4, distribution plots

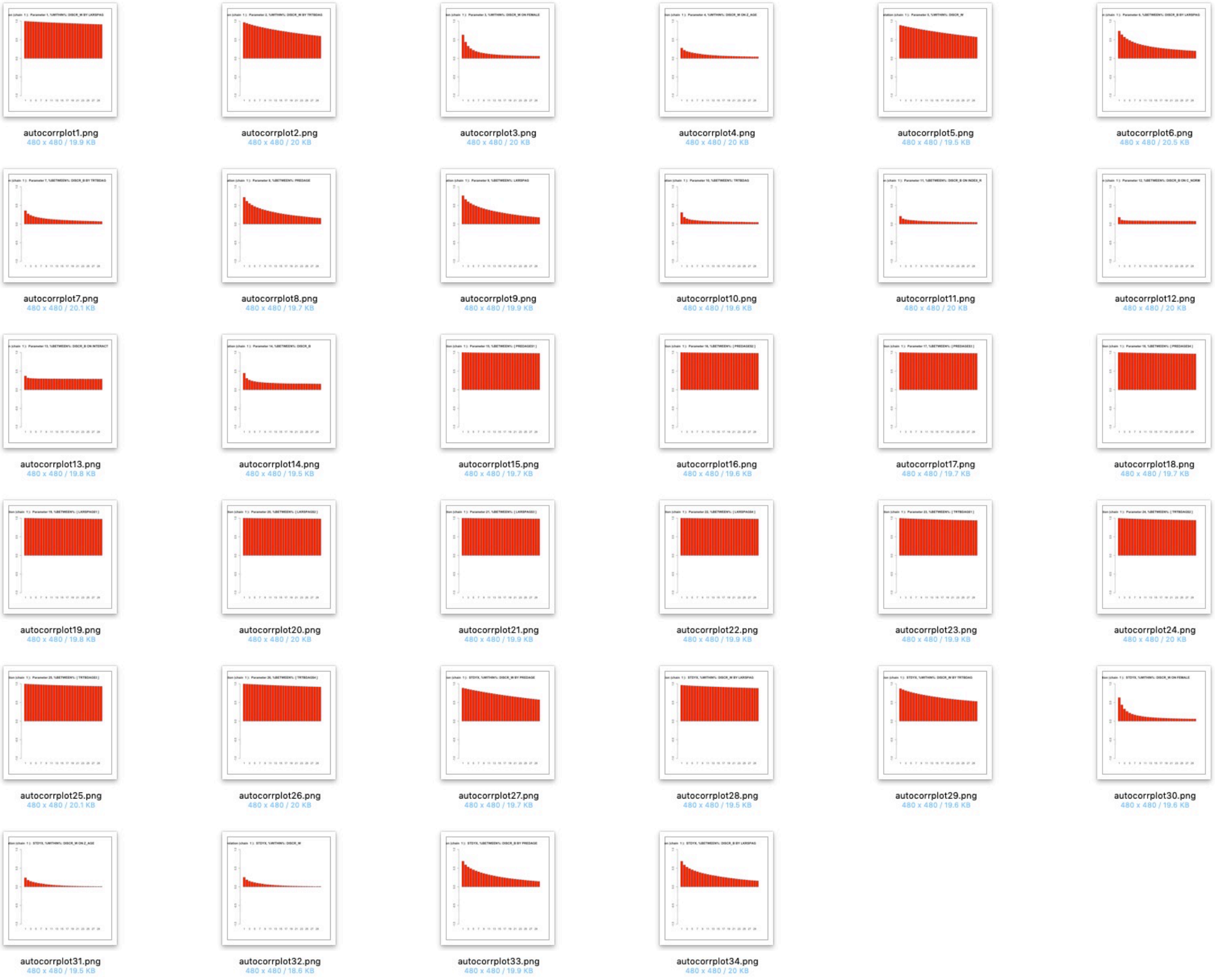

Figure S30: Younger respondents, Model 4, autocorrelation plots

Additional Documentation

Examples of Input for Multilevel SEM

Below are examples of input code for Mplus analyses of a multilevel SEM model with maximum likelihood (first example) and Bayesian estimations (second example).

Some variables were recoded. The three indicators of experiences of age discrimination were recoded (predj_r lkrsp_r trtbd_r), as described in the Measurement section of the article. The variable z_age_young was centered age among younger respondents (aged 15 to 29).

Model 2: Social Norms, Younger Respondents with MLR Estimation

```
TITLE: Younger Respondents Model 2;
DATA:
  FILE = "mplusdata.dat";

VARIABLE:
  NAMES = predj_r lkrsp_r trtbd_r female z_age_young c_norm20 young_old country;
  MISSING=.;
    useobservations is young_old == 0;
    usevariables = predj_r lkrsp_r trtbd_r z_age_young
                   female country c_norm20;
    categorical = predj_r lkrsp_r trtbd_r;
    within = z_age_young female ;
    between = c_norm20;
    cluster=country;
ANALYSIS: type = twolevel;
MODEL:
    %within%
      discrim_w BY predj_r lkrsp_r trtbd_r;
      discrim_w ON z_age_young female;
    %between%
      discrim_b BY predj_r lkrsp_r trtbd_r;
      discrim_b ON c_norm20;
OUTPUT: stdyx; cinterval;
```

Model 2: Social Norms, Younger Respondents with Bayesian Estimation

```
TITLE: Younger respondents Model 2 (Bayes);
DATA:
FILE = "mplusdata.dat";

VARIABLE:
NAMES = predj_r lkrsp_r trtbd_r female z_age_young idxvalue c_norm20 young_old
     country;
 MISSING=.;
    useobservations is young_old == 0;
    usevariables = predj_r lkrsp_r trtbd_r z_age_young
                   female country c_norm20;
    categorical = predj_r lkrsp_r trtbd_r;
    within = z_age_young female ;
    between = c_norm20;
    cluster=country;
ANALYSIS:
    estimator = Bayes; fbiteration = 1500000;
    chains = 2; processors = 8;
    bseed = 1234;
    type = twolevel;
MODEL:
    %within%
      discrim_w BY predj_r lkrsp_r trtbd_r;
      discrim_w ON z_age_young female;
    %between%
      discrim_b BY predj_r lkrsp_r trtbd_r;
      discrim_b ON c_norm20;
OUTPUT: stdyx; tech1; tech8;
PLOT: type = plot2;
```

References

Hallquist, M. N., & Wiley, J. F. (2018). Mplusautomation: An r package for facilitating large-scale latent variable analyses in mplus. Structural Equation Modeling: A Multidisciplinary Journal, 1-18. doi: 10.1080/10705511.2017.1402334

Link, W. A., & Eaton, M. J. (2012). On thinning of chains in MCMC. Methods in Ecology and Evolution, 3(1), 112-115. doi: 0.1111/j.2041-210X.2011.00131.x

Wickham, H. (2009). Ggplot2: Elegant graphics for data analysis. New York: Springer-Verlag.

Xie, Y. (2016). knitr: A General-Purpose Package for Dynamic Report Generation in R. R package version 1.15.1.

Zhu, H. (2019). Package 'kableExtra'. Retrieved from https://cran.r-project.org/web/packages/kableExtra/kableExtra.pdf
